# Phosphatidylcholine with C26:0 moiety, a precursor of a diagnostic marker for X-ALD, is synthesized by LPLAT10/LPEAT2

**DOI:** 10.1016/j.jlr.2025.100973

**Published:** 2025-12-30

**Authors:** Kotaro Hama, Yuko Fujiwara, Koko Imai, Yoshio Kusakabe, Yasuhiro Hayashi, Shigeo Takashima, Shohei Azuma, Masaru Kondo, Atsushi Yamashita, Ryo Takita, Nobuyuki Shimozawa, Kazuaki Yokoyama

**Affiliations:** 1 Faculty of Pharmaceutical Sciences, Teikyo University, Itabashi-ku, Tokyo, Japan; 2 Advanced Comprehensive Research Organization (ACRO), Teikyo University, Itabashi-ku, Tokyo, Japan; 3 School of Pharmaceutical Sciences, University of Shizuoka, Shizuoka, Japan; 4 Division of Genomics Research, Life Science Research Center, Gifu University, Gifu, Japan

**Keywords:** C26:0-lysophosphatidylcholine, fatty acid/transport, glycerophospholipids, lysophospholipid acyltransferase, lipolysis and fatty acid metabolism, newborn screening, peroxisome, phospholipids/metabolism, phospholipids/phosphatidylcholine, very long-chain fatty acid

## Abstract

X-linked adrenoleukodystrophy (X-ALD) is a congenital metabolic disorder characterized mainly by inflammatory demyelination and adrenal insufficiency. Newborn screening using hexacosanoyl lysophosphatidylcholine (C26:0-LPC) in dried blood spots as a diagnostic marker can successfully identify potential patients with X-ALD and prevent disease onset. C26:0-LPC accumulates in patients with X-ALD, although the machinery synthesizing it has remained unclear. In this study, we focused on phosphatidylcholine (PC) with C26:0 moiety as a precursor of C26:0-LPC. We identified that lysophospholipid (LPL) acyltransferase 10 (LPLAT10)/LPCAT4/LPEAT2/AGPAT7 (1-acylglycerol-3-phosphate O-acyltransferase 7) is the responsible LPL acyltransferase that produces PC with C26:0 moiety by transferring C26:0-CoA into 2-acyl-LPC. We also found that LPLAT10 deficiency decreased the amount of C26:0-LPC in fibroblasts from X-ALD patients. Mechanistically, LPLAT10 introduced saturated fatty acid-CoA of various chain lengths as substrates into the *sn*-1 position of LPC but did not transfer C26:0-CoA to other LPL classes, such as lysophosphatidylethanolamine. Structural analysis revealed that a trimethylamine group of PC was placed between two tryptophan residues (W242 and W244), forming a W-X-W motif, possibly through cation-π interaction. Finally, it was shown that exogenously administered C26:0 FFA-*d*_4_ was preferentially incorporated into sphingolipids in the absence of LPLAT10. These results suggest that C26:0-LPC is produced through acyl-chain remodeling of PC catalyzed by LPLAT10 and accumulates in the plasma from X-ALD patients.

X-linked adrenoleukodystrophy (X-ALD) is a congenital metabolic disorder caused by the dysfunction of ATP-binding cassette subfamily type D1 (ABCD1) present on the peroxisomal membrane (1). Very long-chain fatty acyl-CoA (VLCFA-CoA), namely, those with a chain length of 24 or more carbons, are produced through intracellular fatty acid elongation reactions, and most of them are degraded by peroxisomal β-oxidation (2, 3). ABCD1 is essential for transporting VLCFA-CoA into peroxisomes, and thus, very long-chain fatty acids (VLCFAs) and their metabolites accumulate in X-ALD patients (4, 5, 6, 7). However, the causal relationship between the abnormal VLCFA metabolism and the clinical features of X-ALD patients, such as severe inflammatory demyelination and adrenal insufficiency, remains elusive. Hematopoietic stem cell transplantation can delay or halt the progression of this disease but does not recover the disrupted tissues or cells (8). Therefore, early diagnosis and appropriate medical intervention are the most important factors for treating X-ALD.

Newborn mass screening for X-ALD is effective for its early diagnosis. For example, such newborn screening started in New York State in 2013 and is now being conducted in most states of the United States as well as the Netherlands. Pilot screening programs have also started in Italy, Taiwan, and Japan (9, 10, 11, 12, 13). Such screening has revealed a high proportion of null variants of ABCD1 and genetic lineages that potentially contribute to the treatment of X-ALD. However, various issues have been highlighted as causing inaccurate and inefficient diagnosis, including the existence of variants of uncertain significance and the identification of unexpected diseases (Aicardi-Goutières syndrome) in the screening (14, 15). X-ALD screening is based on the amount of lysophosphatidylcholine (C26:0-LPC) in dried blood spots from newborns (16). C26:0-LPC has been found to accumulate not only in the blood but also in the fibroblasts of X-ALD patients and in the blood and organs of ABCD1-KO mice (17, 18, 19). However, it is unclear how C26:0-LPC is produced via the dysfunction of ABCD1.

C26:0-LPC has a structure in which C26:0 is ester bonded to the hydroxyl group at the *sn*-1 position on the glycerol backbone of glycerophosphocholine. Notably, it has been reported that phosphatidylcholine (PC) in which C26:0 is bound to the *sn*-1 position of the glycerol backbone and C16:0, C18:1, C18:2, C20:4, and other PUFAs bound to the *sn*-2 position accumulate in fibroblasts from X-ALD patients and ABCD1-KO mice (18, 20). These results raise the possibility that C26:0-LPC is produced from PC with C26:0 moiety at the *sn*-1 position by the action of various known or as-yet-unidentified phospholipase A_2_. It is thus assumed that the synthetic machinery of PC with C26:0 moiety is important to identify the production of C26:0-LPC in X-ALD patients.

Phospholipids in biological samples exist in a wide variety of molecular forms due to different combinations of polar heads and acyl groups (21). Lysophospholipid (LPL) acyltransferases (LPLATs), which are encoded by 14 genes in humans, are enzymes that introduce acyl-CoA to hydroxyl groups on the glycerol backbone of LPLs (22). Each enzyme displays selectivity in the types of LPL and acyl-CoA as acceptor and donor substrates, respectively, and thus LPLAT enzymes are suggested to be important for the diversity of phospholipid species (23). In this study, we identified an LPLAT that is involved in the production of PC with a C26:0 moiety and confirmed its effect on the amount of C26:0-LPC in fibroblasts from X-ALD patients.

## Materials and Methods

### Ethics

This study was performed in accordance with the tenets of the Declaration of Helsinki, and the research protocol was approved by the Ethics Committee of Teikyo University (#20-075-2).

### Reagents

PC 16:0-*d*_31_/16:0-*d*_31_, PC 17:0/17:0, PC 15:0/18:1-*d*_7_, 1-oleoyl-*d*_7_-2-hydroxy LPC, phosphatidylethanolamine (PE) 15:0/18:1-*d*_7_, PE 17:0/17:0, phosphatidylserine (PS) 15:0/18:1-*d*_7_, PS 17:0/17:0, phosphatidylinositol (PI) 15:0/18:1-*d*_7_, PI 17:0/17:0, phosphatidylglycerol (PG) 15:0/18:1-*d*_7_, PG 17:0/17:0, SM d18:1/18:1-*d*_9_, cholesteryl ester (CE) 18:1-*d*_7_, and triacylglycerol (TG) 17:0-17:1-17:0-*d*_5_ were purchased from Avanti Polar Lipids, Inc (Alabaster, AL). Deuterium-labeled stearic acid (C18:0-*d*_35_-FFA) was purchased from MedChemExpress (Monmouth Junction, NJ). Deuterium-labeled arachidonic acid (C20:4-*d*_8_-FFA) was purchased from Cayman Chemical (Ann Arbor, MI). C30:0-CoA, C28:0-CoA, C26:0-CoA, C24:0-CoA, and C16:0-*d*_4_-CoA were purchased from Avanti Polar Lipids, Inc. Ceramide (Cer) d18:1/16:0-*d*_31_, SM d18:1/16:0-*d*_31_, and lactosyl-Cer d18:1/17:0 were also obtained from Avanti Polar Lipids. Glucosyl-Cer d18:1/16:0-*d*_3_ was obtained from Cayman Chemical. All siRNAs against acyltransferase, except for glycerol 3-phosphate acyltransferase 3 (GPAT3) and LPLAT9, were obtained from Silencer Select RNAi (Thermo Fisher Scientific, Waltham, MA) as listed in Supplemental Table S1, and a control siRNA was obtained from MISSION siRNA Universal Negative Control (Sigma-Aldrich, St Louis, MO). Two siRNAs against GPAT3 and LPLAT2 were kindly gifted by Dr Kazunari Tanigawa (Teikyo University). The lentiCRISPRv2 and psPAX2 vectors were gifts from Dr Feng Zhang (Massachusetts Institute of Technology, Cambridge, MA) and Dr Didier Trono (Ecole Polytechnique Fédérale de Lausanne, Lausanne, Switzerland), respectively. Both plasmids were obtained through Addgene (Watertown, MA). All chemicals used in the mobile phases were purchased from Fujifilm Wako Pure Chemical Corporation (Osaka, Japan). Methyl-β-cyclodextrin (MβCD) was obtained from Sigma-Aldrich, dissolved in water (50 mM), and stored at −20°C. Organic solvents and reagents used in the synthesis of C26:0 FFA-*d*_4_ were purchased from commercial suppliers and used without further purification: tetrahydrofuran (THF; 99.5%; Kanto Chemical Co, Inc [Chuo-ku, Japan]), toluene (99.5%; Kanto Chemical Co, Inc), dichloromethane (99.5%; Kanto Chemical Co, Inc), methanol-*d*_1_ (99.5% D; Tokyo Chemical Industry Co, Ltd), deuterium oxide (99.9% D; Eurisotop), C26:0 FFA (cerotic acid, 95%; Tokyo Chemical Industry Co, Ltd), 8-aminoquinoline (98%; Tokyo Chemical Industry Co, Ltd), 1-ethyl-3-(3-dimethylaminopropyl)carbodiimide hydrochloride (Peptide Institute, Inc), 4-dimethylaminopyridine (99%; Tokyo Chemical Industry Co, Ltd), palladium acetate (97%; Fujifilm Wako Pure Chemical Corporation), cesium pivalate (98%; Sigma-Aldrich), potassium carbonate (99.5%; Fujifilm Wako Pure Chemical Corporation), nickel(II) bis(2,2,6,6-tetramethyl-3,5-heptanedionate) (97%; Sigma-Aldrich), and sodium deuteroxide (40 wt. % in D_2_O, 99.5 atom % D; Sigma-Aldrich).

### Generation of LPLAT- and GPAT-KO HeLa cells with the CRISPR/Cas9 system

To establish each of the LPLAT-KO and GPAT-KO cell lines, we designed guide RNAs for each gene using the web-based CRISPR design tool CRISPOR (https://crispor.gi.ucsc.edu) (24). The oligonucleotide sequences for the guide RNA of each gene are listed in Supplemental Table S2. Plasmid construction was performed as previously described (25). Briefly, oligonucleotide pairs for the guide RNA of each gene were cloned into the pSpCas9(BB)-2A-GFP (PX458) vector. The constructs were transfected into HeLa cells using Lipofectamine 2000 (Thermo Fisher Scientific), in accordance with the manufacturer’s instructions. Enhanced GFP-positive cells were collected with a FACSAria III (BD Biosciences, Franklin Lakes, NJ), and clonal populations were obtained by limiting dilution. The deficiency of each gene was screened by a PCR-restriction enzyme (PCR-RE) assay. The oligonucleotide sequences and the restriction enzymes used in the PCR-RE assay for each gene are listed in Supplemental Table S3. The obtained PCR products were subcloned into a T-vector (pMD20; Takara Bio, Inc, Otsu, Japan) and sequenced to confirm the mutations in each KO cell line. To introduce mutations into fibroblasts from X-ALD patients, oligonucleotide pairs for LPLAT10 were cloned into the lentiCRISPRv2 vector as previously described (26). The obtained construct was cotransfected with the psPAX2 plasmid and a vector encoding envelope protein (vesicular stomatitis virus glycoprotein) into Lenti-X 293T cells (Takara Bio) using Lipofectamine 2000 (Thermo Fisher Scientific), in accordance with the manufacturer’s instructions. Two days later, the conditioned medium containing virus particles was harvested, mixed with polybrene (Nacalai Tesque, Kyoto, Japan) at a concentration of 4 μg/ml, and immediately used for lentiviral infection of fibroblasts overnight. After 24 h, the cells were screened using puromycin (2 μg mL^−1^), and the KO efficiency was validated by a PCR-RE assay. The oligonucleotide sequence and the restriction enzymes used to generate LPLAT10-KO fibroblasts were identical to those used to confer LPLAT10 deficiency on HeLa cells.

### Cell lines and sample preparation

Primary human fibroblasts were established from skin biopsy samples of three patients with X-ALD. HeLa cells were obtained from the cell bank of Riken Bioresource Center (Ibaraki, Japan). ABCD1-KO HeLa cell lines were previously established (20). Human skin fibroblasts and HeLa cells were cultured in minimum essential medium (Sigma-Aldrich) supplemented with 10% FBS (Biowest, Nuaillé, France), 2 mM l-glutamine (Thermo Fisher Scientific), 100 U mL^−1^ penicillin, and 100 μg mL^−1^ streptomycin (Sigma-Aldrich). HeLa cells (2.0 × 10^6^ cells per 10 cm culture dish) and fibroblasts (5.0 × 10^5^ cells per 10 cm culture dish) were seeded and cultured overnight in medium containing 10% FBS, followed by lipid extraction. Prior to siRNA-based screening or metabolic labeling experiments, C26:0 FFA or C26:0 FFA-*d*_4_ was mixed with MβCD to form FFA-MβCD complexes, as described previously (27). Then, the cells were treated with 30 μM FFA-MβCD complexes in medium containing 10% FBS for 24 h, followed by lipid extraction.

### Lipid extraction

For the analyses of FFA, phospholipids (PC, PE, PS, PI, PG, and SM), TG, CE, and total lipid fractions were extracted from cells by the Bligh and Dyer methods, as previously reported (20, 28). Sample preparations for Cer, monohexosylceramide (mono-HexCer), and dihexosylceramide (di-HexCer) were prepared as described previously (29). Protein concentrations of cell homogenates were determined using a BCA protein assay kit (Thermo Fisher Scientific).

### LC-MS/MS analysis

Each phospholipid, LPL, acyl-CoA, FFA, TG, CE, and sphingolipid species, including Cer, mono-HexCer, and di-HexCer, was analyzed using a QTRAP 4500 (Sciex, Framingham, MA) linked to a Nexera XR HPLC system (Shimadzu Corp, Kyoto, Japan), as described previously (18, 20, 28, 29). A CAPCELL PAK C_18_ ACR column (1.5 mm i.d. × 100 mm, particle size 3.0 μm; OSAKA SODA, Co, Ltd) was used at 50°C.

**Supplemental Fig. 1:**
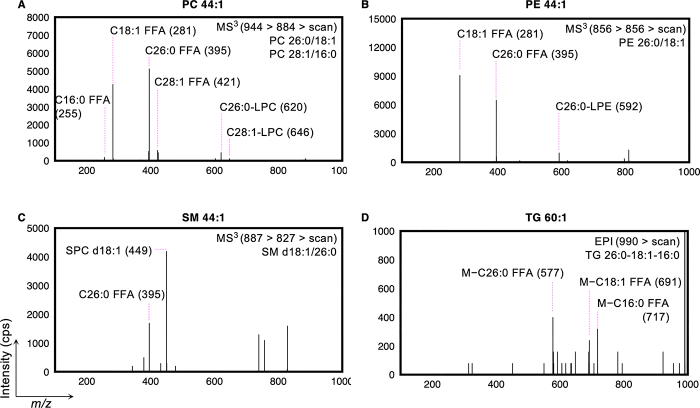


The composition, programmed solvent gradient, and flow rate of mobile phases are listed in Supplemental Table S4. The conditions of ionization and the transitions of selected reaction monitoring are listed in Supplemental Tables S5–S8. The structural analyses of the PC 44:1, PE 44:1, SM 44:1, and TG 60:1 species were conducted with the LC-MS^3^ or enhanced product ion method as previously described to determine the fatty acyl moieties in each lipid species (Supplemental Fig. S1) (28). The quantitation of each lipid species was performed using the internal standard method. For instance, 50 pmol of PC 15:0/18:1-*d*_7_ was added to the sample, and the total lipid fraction was obtained. Quantitation was based on the area ratio of the chromatogram for PC 15:0/18:1-*d*_7_ and each PC species. This quantitative method complies with level 2 of the Lipid Standards Initiative (https://lipidomicstandards.org/lipid-species-quantification/) as defined by matching the internal standard to the lipid class of the analyte and coionization of the analyte and internal standard. Analyst software and MultiQuant software (Sciex) were used for data acquisition and data processing.

### In vitro acyltransferase assay

The LPLAT activity was determined as previously described (30). Human embryonic kidney 293T cells transiently expressing each LPLAT gene were harvested and homogenized using an ultrasonic homogenizer. Membrane fractions were obtained by ultracentrifugation at 100,000 g and 4°C for 1 h, were resuspended in buffer (20 mM Tris-HCl [pH 7.4], 300 mM sucrose, and 1 mM EDTA), and then the protein concentrations were determined using a Bradford protein assay (Bio-Rad Laboratories, Richmond, CA). The assay was conducted in reaction buffer (110 mM Tris-HCl [pH 7.4], 150 mM sucrose, 0.5 mM EDTA, and 0.015% Tween-20) containing 10 μM acyl-CoA and 20 μM LPL as donor and acceptor substrates, respectively. The reaction was initiated by adding 1 μg of membrane protein. After incubation at 37°C for 10 min, the reaction was terminated by adding CHCl_3_-MeOH (1:2, v/v). The total lipid fraction was extracted by the Bligh and Dyer method and analyzed by LC-MS/MS.

### Molecular docking simulation

To examine the binding mode of LPLAT10-PC 26:0/16:0 complex, we conducted docking experiments using AutoDock Vina (Scripps Research, La Jolla, CA), as previously described (31). The 3D structure of full-length human LPLAT10 (AF-Q643-F1-v4) was obtained from AlphaFold2 (32, 33, 34). Hydrogen atoms and AM1-BCC atomic charge were added to PC 26:0/16:0 using the *Hgene* program in the *myPresto* portal (35). Then, energy optimization to generate 3D conformations of PC 26:0/16:0 was conducted using the *cofgene* program in the *myPresto* portal. For all docking simulations, box centers were set to the default settings. To include all LPLAT10 structures, a grid box size of 60 × 60 × 60 Å against the monomeric structure of LPLAT10 and exhaustiveness of 100 were used as docking parameters (36, 37). The spacing between grid points was adjusted to 1 Å, and the maximum number of docking modes was set to 20. The docking poses were visualized using the PyMOL Molecular Graphics System (version 3.0; Schrödinger, LLC).

### Molecular dynamics simulation procedures

The LPLAT10-PC26:0/16:0 complexes with the highest binding affinities (kcal/mol) based on the docking simulations performed in AutoDock Vina (Scripps Research) were used as the starting structures for molecular dynamics (MD) simulations (31). First, LPLAT10-PC26:0/16:0 complex was immersed in a transferable intermolecular potential 3P water box, with water molecules extending 15.0 Å from any solute atom in each direction using Bondi radii. Then, Na^+^ or Cl^−^ ions were added to neutralize each complex. The complexes were minimized in two steps. After all the heavy backbone atoms of LPLAT10 were restrained with a weight of 2 kcal/mol Å^2^, the system was minimized without any restraints. These systems were optimized by 5,000 cycles of steepest descent and 5,000 cycles of conjugate gradient minimization. All minimizations were performed using the *sander* Amber package. Following minimization, the Amber 24 simulation package was used to carry out MD simulations (38, 39). The total simulation time for PC 26:0/16:0 and LPLAT10 protein complexes was 100 ns, in which the complexes were heated from 0 to 300 K at 25 ps, in constant volume mode with a restraint weight of 2.0 kcal/mol Å^2^; then, density balance was carried out with a restraint weight of 2.0 kcal/mol Å^2^ (constant pressure of 10 ps), equilibrium (constant pressure of 100 ps). During 100 ns MD calculations, the docking poses with the lowest energy were defined as the binding models.

### Synthesis of acyl-CoA species

Deuterium-labeled stearoyl-CoA (C18:0-*d*_35_-CoA) and arachidonoyl-CoA (C20:4-*d*_8_-CoA), as well as C20:0-CoA and C22:0-CoA, were synthesized as described previously, with slight modifications (20). Briefly, 8.6 μmol of C18:0-*d*_35_-FFA, C20:0-FFA, or C22:0-FFA and 2 ml of oxalyl chloride were mixed by stirring overnight at room temperature to yield acyl chlorides. Excess oxalyl chloride was removed with nitrogen gas, and the oil residue of fatty acid chloride was dissolved in 0.5 ml of freshly distilled THF. The acid chloride solution was added slowly to a CoA solution (7.5 mg of CoA dissolved in 2 ml of THF/0.1 M Tris-HCl, pH 7.4 [2/1 by volume]) in a 10 ml screw-capped test tube to maintain the pH at approximately 8 by using 1 M NaOH. After the addition of the acid chloride solution, each acyl-CoA was purified with 2-(2-pyridyl)ethyl silica gel columns (Sigma-Aldrich) using elution buffer (MeOH/H_2_O = 80/15 supplemented with 50 mM ammonium formate and 0.112% ammonia). A total of 0.86 μmol C20:4-*d*_8_-FFA and 0.75 mg of CoA were used to obtain the C20:4-*d*_8_-CoA. The obtained acyl-CoA species were reconstituted with methanol and stored at −20°C.

### Preparation of LPLs

Two forms of 1-acyl- and 2-acyl-LPLs were prepared by PLA_2_ and PLA_1_ reactions, as previously described, with slight modifications (40, 41). To obtain 2–16:0-*d*_31_-LPC, 0.2 μmol PC 16:0-*d*_31_/16:0-*d*_31_ was incubated for 16 h at 4°C under vigorous stirring in 200 μl of PLA_1_ buffer (30 mM CH_3_COONa, 3.7 mM CH_3_COOH, 6.7 mM CaCl_2_, and 68 mM NaCl, and 5 μl of PLA_1_ solution containing approximately 150 units of a lipase from *Rhizopus oryzae* [Sigma-Aldrich]). The reaction was terminated by adding 1.35 ml of methanol containing 10 mM ammonium formate and 0.1% formic acid (v/v), followed by solid-phase extraction using an Oasis HLB column (Waters, Milford, MA), in accordance with the manufacturer’s instructions. The eluent in methanol containing 10 mM ammonium formate and 0.1% formic acid (v/v) was evaporated using an EZ-2 centrifugal evaporator (Genevac). The resulting precipitate was reconstituted with 0.1 ml of methanol and immediately used for lipid analysis by LC-MS. To obtain 1-16:0-*d*_31_-LPC, 0.2 μmol PC 16:0-*d*_31_/16:0-*d*_31_ was incubated for 16 h at 4°C under vigorous stirring in 2000 μl of PLA_2_ buffer (50 mM Tris-HCl [pH 8.0], 25 mM CaCl_2_, 0.1% Triton X-100, 3.7 mM CH_3_COOH, 6.7 mM CaCl_2_, 68 mM NaCl, and 5 μl of PLA_2_ containing approximately 150 units of a lipase from *Apis mellifera* [Cayman Chemical]). The LPL fraction was purified by solid-phase extraction using an Oasis HLB column. Here, 1- (or 2-)17:0-LPC, lysophosphatidylethanolamine (LPE), lysophosphatidylserine, lysophosphatidylinositol, and lysophosphatidylglycerol were obtained from corresponding PC, PE, PS, PI, and PG harboring two 17:0-moieties by the PLA_2_ or PLA_1_ reaction, respectively.

### Synthesis of C26:0 FFA-*d*_4_

C26:0 FFA-*d*_4_ was synthesized by the following modified procedure based on our previously reported α,β-selective tetradeuteration process of straight-chain fatty acids (30). The detailed procedures for synthesis are described in the supplemental data.

### Statistical methods

Statistical analysis was performed either by one-way ANOVA, followed by Dunnett's T3 or Tukey’s post hoc test, or by Student’s *t-*test. Differences were considered to be significant if the *P* value was <0.05. All statistical analyses were conducted with IBM SPSS Statistics, version 23 (IBM, Armonk, NY).

## Results

### LPLAT10 is indispensable for the accumulation of PC with C26:0 moiety in ABCD1-KO HeLa cells

**Fig. 1:**
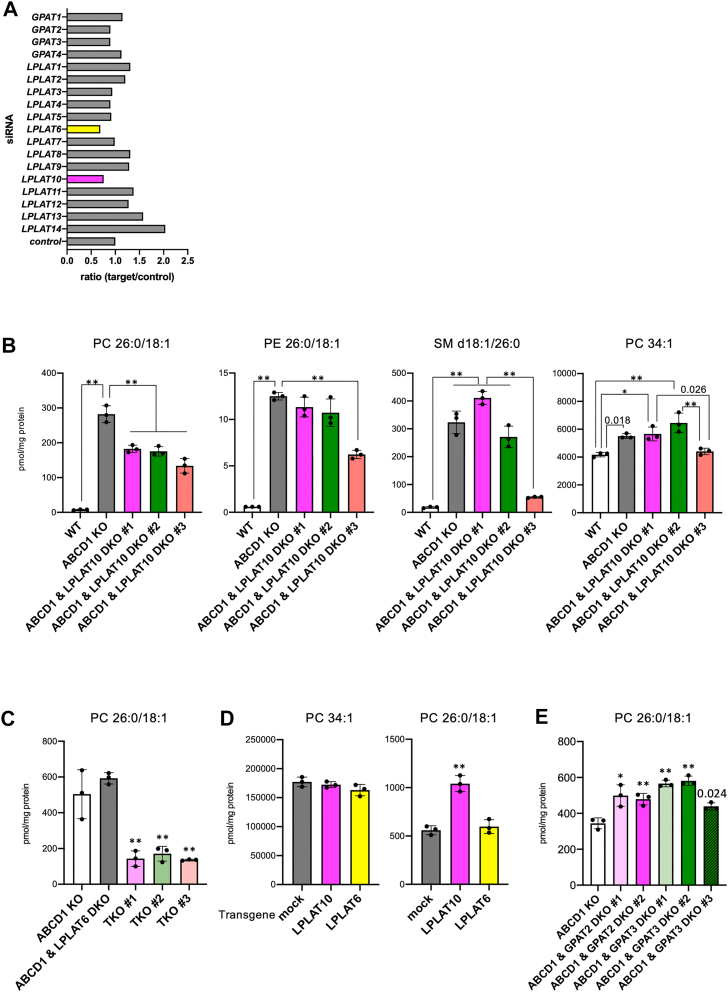
Identification of an acyltransferase involved in the synthesis of VLCFA-PL. A: Screening of GPATs and LPLATs involved in the synthesis of PC species with a VLCFA moiety. ABCD1-deficient HeLa cells were incubated with free C26:0 FFA for 24 h. The amounts of three PC species (PC 42:0, PC 44:1, and PC 44:2) with C26:0 moiety were analyzed by LC-MS/MS. B: The amounts of phospholipid species in ABCD1 and LPLAT10-DKO HeLa cells. C: The amounts of PC species in ABCD1 and LPLAT6-DKO and ABCD1, LPLAT6, and LPLAT10-TKO HeLa cells. D: The amounts of PC species in ABCD1 and LPLAT10-DKO HeLa cells transfected with the LPLAT10 or LPLAT6 gene. E: The amounts of PC 44:1 in ABCD1 and GPAT2- (#1 and 2) or ABCD1 and GPAT3-DKO HeLa cells (#1–3). Note that each of the PC, PE, and SM species was analyzed quantitatively in positive ion mode, followed by MS^3^ analysis to obtain structural insights, including into acyl moieties in each phospholipid (e.g., Supplemental Fig. S2). Data represent the mean ± SD; statistical analysis was performed using one-way ANOVA, followed by Tukey's post hoc test (B) or Dunnett's test (vs. ABCD1 KO in C and E or vs. mock in D). *∗P* < 0.01, *∗∗P* < 0.001.

**Supplemental Fig. 2:**
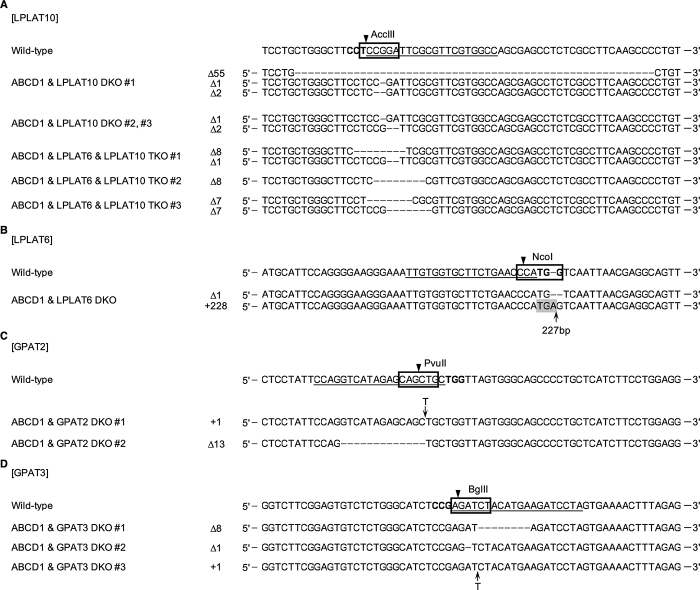


To identify the LPLAT involved in the production of PC with C26:0 moiety, we used ABCD1-KO HeLa cells, which were previously established (20). The cells were treated with each LPLAT siRNA and incubated with C26:0 FFA. The total lipid fraction was extracted from the cells 24 h later, given the previous observation that most exogenously administered C26:0 FFA is gradually transformed into C26:0-CoA over at least 24 h after addition (20). We analyzed the three PC species (PC 42:0, PC 44:1, and PC 44:2) with C26:0 moiety (e.g., PC 26:0-16:0 in PC 42:0) and observed approximately a 30% reduction in their total amounts in cells treated with LPLAT6/LCLAT1 or LPLAT10 siRNA (Fig. 1A). Then, we disrupted the *LPLAT6* and *LPLAT10* genes in ABCD1-KO cells and verified the contribution of each LPLAT to the production of PC with C26:0 moiety (Fig. S2A, B). The amount of PC with C26:0 moiety (PC 26:0/18:1) was significantly reduced in three independent clones of ABCD1 and LPLAT10-double KO (DKO) cells compared with that of ABCD1-KO cells, whereas the amount of PE and SM species with C26:0 moiety (PE 26:0/18:1 and SM d18:1/26:0) was not significantly altered in two of the three DKO clones (Fig. 1B). Note that the amount of PC 34:1 consisting of long-chain fatty-acyl moieties was significantly decreased in the clone of ABCD1 and LPLAT10-DKO #3, suggesting that the overall process of phospholipid synthesis was affected for some reason (Fig. 1B). The amount of PC with C26:0 moiety (PC 26:0/18:1) was not significantly changed in ABCD1 and LPLAT6-DKO cells compared with that of ABCD1-KO cells but was significantly decreased by modifying the *LPLAT10* gene in ABCD1- and LPLAT6-DKO cells compared with that of ABCD1-KO cells (Fig. 1C). Furthermore, the amount of PC with C26:0 moiety (PC 26:0/18:1) was significantly increased by exogenously expressing LPLAT10, but not by expressing LPLAT6, in ABCD1 and LPLAT10-DKO cells (Fig. 1D). We previously observed that a GPAT that produces lysophosphatidic acid and LPLAT10 cooperatively contribute to the synthesis of PC with hydroxyoctadecadienoic acyl moiety (30). When we treated ABCD1-KO HeLa cells with each GPAT siRNA and then performed incubation with C26:0 FFA, the amount of PC with C26:0 moiety species (PC 42:0, PC 44:1, and PC 44:2) was slightly decreased in cells treated with GPAT2 and GPAT3 siRNA (Fig. 1A). In ABCD1 and GPAT2- or ABCD1 and GPAT3-DKO cells, however, the amount of PC with C26:0 moiety did not significantly decrease but rather increased (Fig. 1E, Supplemental Fig. S2C, D). Taking these findings together, we concluded that LPLAT10 is indispensable for synthesizing PC with the C26:0 moiety in ABCD1-KO HeLa cells.

### LPLAT10 is indispensable for the accumulation of VLCFA-PC in fibroblasts from X-ALD patients

**Fig. 2:**
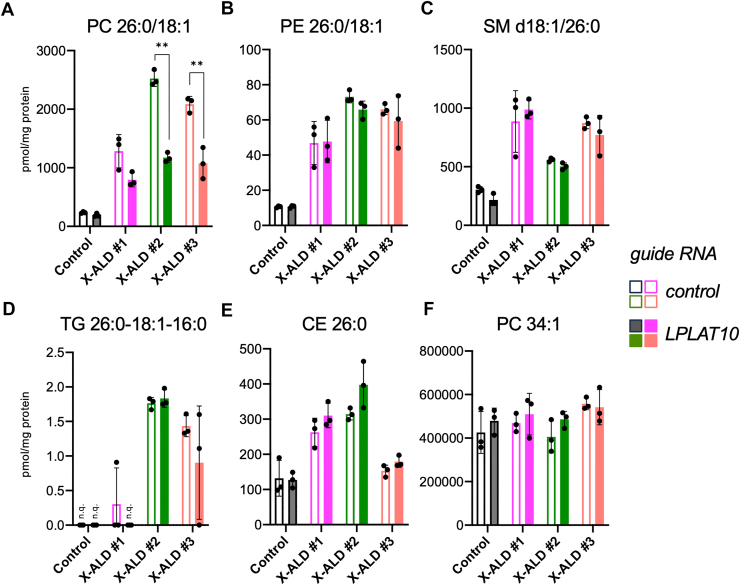
PC with C26:0 moiety was selectively altered in LPLAT10-KO fibroblasts from X-ALD patients. The amount of PC (A), PE (B), SM (C), TG (D), and CE (E) species with C26:0-moiety as well as PC 34:1 (F) in fibroblasts. Fibroblasts from X-ALD patients were infected with lentivirus-expressing guide RNA targeting the *LPLAT10* gene. Each of the lipid species in the total lipid fractions was analyzed by LC-MS/MS. Note that each of the PC, PE, SM, and TG species was analyzed quantitatively in positive ion mode, followed by MS^3^ analysis to obtain structural insights including into acyl moieties in each phospholipid or TG (e.g., Supplemental Fig. S2). Data represent the mean ± SD; statistical analysis was performed using Student’s *t-*test. *∗∗P* < 0.001.

**Supplemental Fig. 3:**
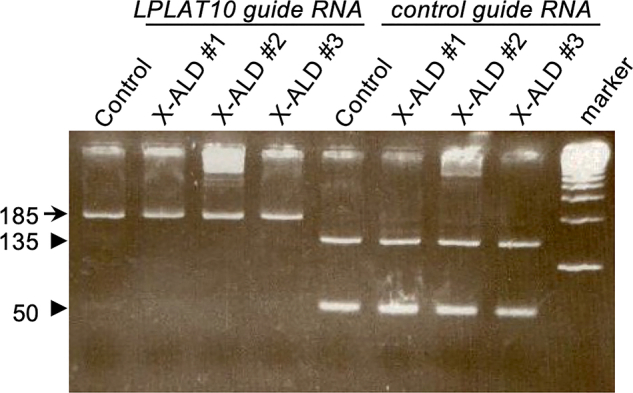


We used fibroblasts derived from X-ALD patients to examine the contribution of LPLAT10 to the synthesis of PC with C26:0 moiety. Primary cultured fibroblasts have limited proliferative capacity compared with HeLa cells, so we applied a genome editing method using lentivirus, which is expected to have high gene transfer efficiency. When guide RNA against LPLAT10 was expressed in patient-derived fibroblasts, the PCR product corresponding to the genomic region covering target sequences was resistant to a restriction enzyme (AccIII), confirming the introduction of mutations in the *LPLAT10* gene (Supplemental Fig. S3). The amount of PC with C26:0 moiety, such as PC 26:0/18:1, was significantly reduced in two lines of X-ALD fibroblasts (X-ALD #2 and #3) expressing *LPLAT10* guide RNA, whereas a decreasing trend was observed in the remaining line (X-ALD #1) (Fig. 2A, Supplemental Table S9). By contrast, the amounts of PE, SM, TG, and CE species with C26:0 moiety (PE 26:0/18:1, SM d18:1/26:0, TG 26:0-18:1-16:0, and CE 26:0), as well as PC with long-chain fatty-acyl moiety (PC 34:1), were not significantly altered in the X-ALD fibroblasts expressing *LPLAT10* guide RNA (Fig. 2B–F, Supplemental Table S9). These results showed that LPLAT10 is also indispensable for synthesizing PC with the C26:0 moiety in fibroblasts from X-ALD patients.

### LPLAT10 contributes to the accumulation of C26:0-LPC in fibroblasts from X-ALD patients

**Fig. 3:**
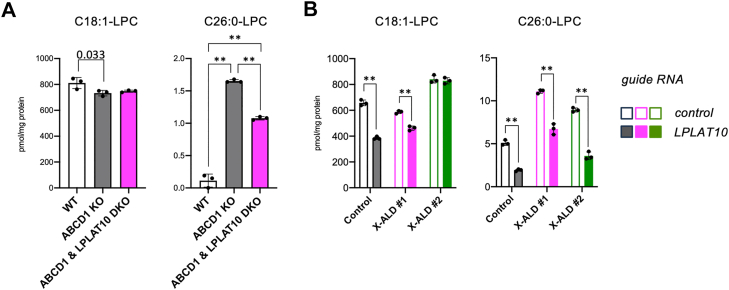
The amounts of LPC species in LPLAT10-KO cells. A: The amounts of LPC with a C18:1- or C26:0 moiety in ABCD1 and LPLAT10-DKO cells. B: The amounts of C18:1-LPC and C26:0-LPC in fibroblasts from two X-ALD patients (#1 and 2) infected with lentivirus-expressing guide RNA targeting the *LPLAT10* gene. Data represent the mean ± SD; statistical analysis was performed using one-way ANOVA, followed by the Tukey's post hoc test (A) or Student’s *t-*test (B). *∗∗P* < 0.001.

PC with C26:0 moiety produced within cells is expected to be converted into C26:0-LPC by intracellular phospholipase A. Therefore, we examined the contribution of LPLAT10 to intracellular C26:0-LPC production. As expected, a significant reduction was observed in the amount of C26:0-LPC, but not in C18:1-LPC, in ABCD1- and LPLAT10-DKO HeLa cells, compared with that of ABCD1-KO cells (Fig. 3A). The amount of C26:0-LPC was significantly decreased not only in X-ALD fibroblasts but also in control fibroblasts expressing *LPLAT10* guide RNA (Fig. 1B). Note that the amount of C18:1-LPC was also decreased in control and X-ALD fibroblast cells (X-ALD #1), suggesting that LPLAT10 could be involved in the production of phospholipids with C18:1 moiety (Fig. 3B). These results revealed that LPLAT10 plays essential roles in the production of intracellular C26:0-LPC in HeLa cells and X-ALD fibroblasts.

### LPLAT10 introduces saturated VLCFA-CoA into 2-acyl-LPC

**Fig. 4:**
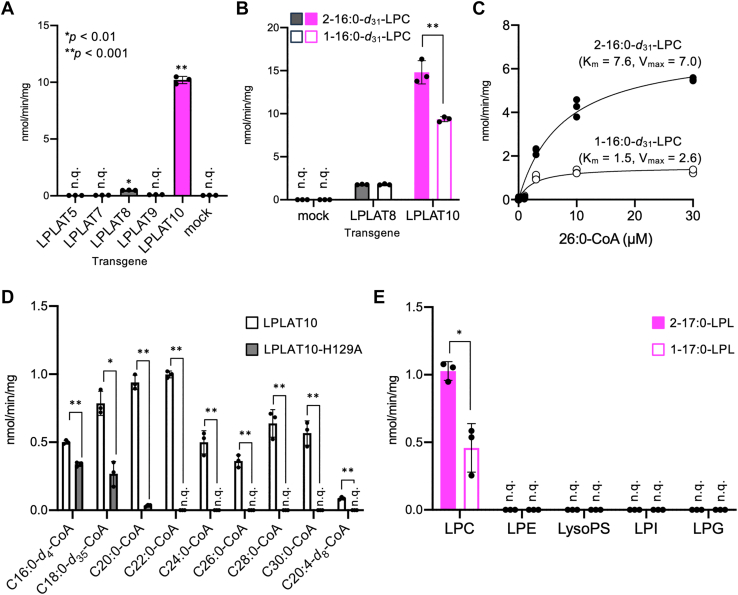
Substrate selectivity of LPLAT10 in vitro. A: LPLAT activity toward C26:0-CoA and 2–16:0-*d*_31_-LPC as donor and acceptor substrates, respectively. Membrane fractions were obtained from human embryonic kidney 293 cells transfected with each *LPLAT* gene. B: LPLAT activity toward 1-16:0-*d*_31_-LPC or 2–16:0-*d*_31_-LPC as acceptor substrates. C: Kinetic analysis of the LPLAT activity of LPLAT10 toward C26:0-CoA and 1-16:0-*d*_31_-LPC or 2–16:0-*d*_31_-LPC was conducted to determine the value of the apparent *K*_*m*_ (nM) and *V*_max_ (nmol/min/mg). D: LPLAT activity toward saturated fatty acyl-CoA and C20:4-CoA as donor substrates. 2–16:0-*d*_31_-LPC was used as an acceptor substrate. E: LPLAT activity of LPLAT10 toward 1-17:0-LPLs or 2-17:0-LPL as acceptor substrates. Data represent the mean ± SD; statistical analysis was performed using one-way ANOVA, followed by the Dunnett's test (vs. mock) in A or Student’s *t-*test in B–E. *∗P* < 0.01 and *∗∗P* < 0.001.

The 14 types of LPLAT (LPLAT1–14) have been reported to display different substrate specificities, contributing to the production of diverse phospholipid species (23). Here, we focused on the substrate specificity of LPLAT10, particularly the acyl transfer reaction of VLCFAs. In previous studies and the present one, it was revealed that VLCFAs are ester-bonded to the hydroxyl group at the *sn*-1 position on the glycerol backbone of phospholipids (18). As such, we prepared 2–16:0-*d*_31_-LPC with phospholipase A_1_ (*Aspergillus oryzae*) as the acceptor molecule to verify the acyl transfer activity of C26:0-CoA (18). We observed significant C26:0-CoA transfer activity in cell membrane fractions expressing LPLAT10 (Fig. 4A). In contrast, LPLAT8/LPCAT1, which has very high homology with LPLAT10 in terms of amino acid sequence, showed slight activity, whereas no activity was observed in LPLAT9/LPCAT2 (Fig. 4A) (42, 43). In addition, the acyl transfer activity was not detected for LPLAT5/AGPAT5 (1-acylglycerol-3-phosphate O-acyltransferase 5) or LPLAT7/LPGAT1, the latter of which has been reported to introduce stearic acid into the *sn*-1 position on the glycerol backbone of phospholipids (Fig. 4A) (44, 45). These results show that LPLAT10 efficiently transfers C26:0-CoA into 2-acyl-LPC. Next, we prepared 1-16:0-*d*_31_-LPC with phospholipase A_2_ (*Apis mellifera*) and compared it with 2-16:0-*d*_31_-LPC to examine which of 1-acyl-LPC and 2-acyl-LPC is preferred as an acceptor substrate. We observed that LPLAT10 efficiently introduced C26:0-CoA into 2-acyl-LPC compared with the case for 1-acyl-LPC (Fig. 4B, C). By contrast, C26:0-CoA transfer activity was not significantly different between 2-acyl-LPC and 1-acyl-LPC when using the membrane fraction expressing LPLAT8 as an enzyme source (Fig. 4B). We further examined the substrate selectivity of LPLAT10 for saturated fatty acyl-CoA of various chain lengths. Significant acyl transfer activity was observed when using any of the tested saturated fatty acyl-CoA from C_16_ (palmitoyl-CoA) to C_30_ (triacontanoyl-CoA) (Fig. 4D). In contrast, the activity toward C20:0 to C30:0-CoA was almost or completely lost for membrane fractions expressing LPLAT10 with a mutation at the active site (H129A) (Fig. 4D). Notably, compared with wild-type LPLAT10, approximately two-thirds or one-third of the activity toward C16:0-*d*_4_-CoA and C18:0-*d*_35_-CoA was observed in membrane fractions expressing the LPLAT10 mutant (H129A), suggesting that LPLAT other than LPLAT10 endogenously expressed in the membrane fraction recognized these long-chain fatty acyl-CoA as substrates (Fig. 4D). It has been reported that LPLAT8/LPCAT1 and LPLAT7/LPGAT1 possess the activity of introducing C16:0 and C18:0 into 2-acyl LPC as the acceptor molecule, respectively (40, 44, 45). In fact, we previously confirmed the expression of both the LPLAT7 and LPLAT8 genes in the human embryonic kidney 293 cells used in this study (30). Based on the above, it is possible that these two LPLATs in the membrane fraction expressing mutant LPLAT10 recognized C16:0-*d*_4_-CoA and C18:0-*d*_35_-CoA. We also confirmed that LPLAT10 transferred C20:4-*d*_8_-CoA into LPC, as reported previously (Fig. 4D) (46). In the present analysis using HeLa cells and X-ALD fibroblasts, the effects of LPLAT10 deficiency were limited to PC species, and no effects were observed on other phospholipid classes such as PE and SM (Figs. 1B and 2). Therefore, we examined the substrate selectivity of LPLAT10 toward each of the 2-17:0- and 1-17:0-LPLs as acceptor molecules. The C26:0-CoA transfer activity of LPLAT10 was detected exclusively toward LPC as acceptor substrates, but not for other LPLs, regardless of whether they were of the 1-acyl or 2-acyl type (Fig. 4E). Taking these results together, the acyl transfer activity of LPLAT10 displays two features: *i*) LPLAT10 can recognize saturated fatty acyl-CoA with a wide range of chain lengths and *ii*) LPLAT10 transfers C26:0-CoA into 2-acyl-LPC in a selective manner.

### 2-acyl-LPC is potentially recognized by W-X-W motif commonly conserved among LPLAT8, LPLAT9, and LPLAT10 belonging to the AYTL family

**Fig. 5:**
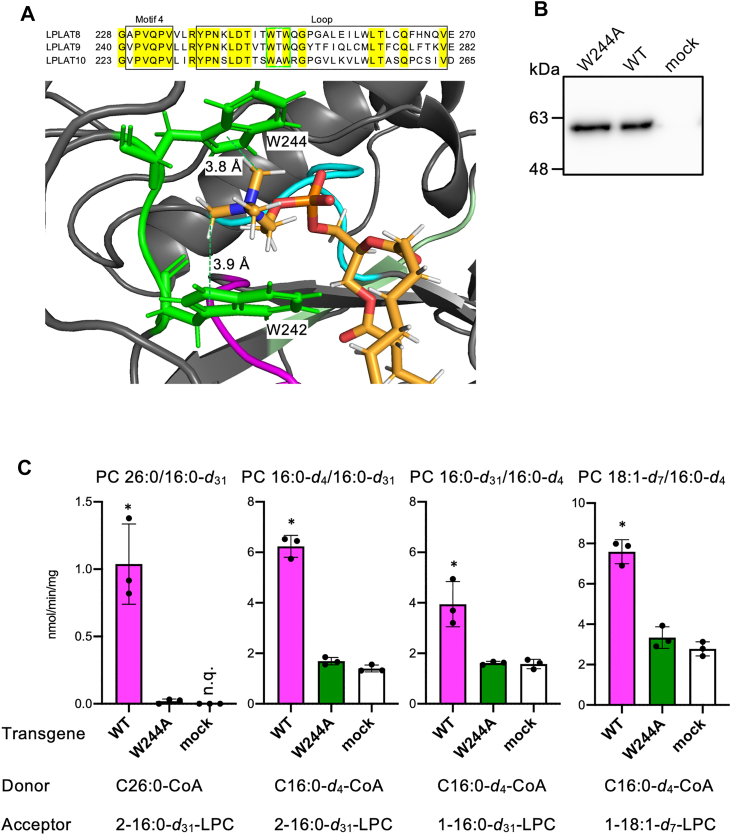
Spatial configuration of the choline group of PC in LPLAT10. A: Multiple sequence alignment of human LPLAT8–10 and docking simulations of LPLAT10 and C26:0-PC. The AGPAT motif 4 and loop domain are represented in a box, whereas the W-X-W motif is represented in a neon-green box. Conserved residues are highlighted in yellow. Spatial relationship between the choline group of PC 26:0/16:0 and two tryptophan residues (W242 and W244) in LPLAT10. Magenta (motif 1, residues 129–134); cyan (motif 3, residues 201–206); yellow-green (motif 4, residues 224–230); and neon-green (W-X-W motif, residues 242–244). The bound PC 26:0/16:0 is shown as orange sticks. B: Immunoblotting analysis of LPLAT10 mutant. The membrane fractions expressing the wild-type (WT) and LPLAT10 mutant (W244A) were analyzed by Western blotting with anti-FLAG antibody. C: LPLAT activity of LPLAT10 mutant (W244A). LPLAT activity was analyzed using 1-acyl or 2-acyl-LPC as acceptor substrates. Data represent the mean ± SD; statistical analysis was performed using one-way ANOVA, followed by the Dunnett's test (vs. mock). *∗P* < 0.01. AGPAT, 1-acylglycerol-3-phosphate O-acyltransferase.

**Supplemental Fig. 4:**
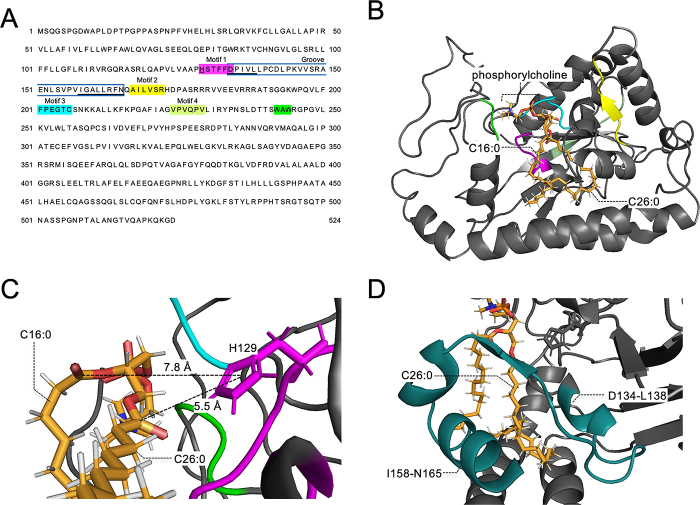


Human LPLAT10 was originally discovered as an LPE acyltransferase (and named LPEAT2), which introduces C18:1-CoA into LPE (47). In a subsequent analysis, it was also shown that mouse Lplat10 transfers C20:4-CoA and C22:6-CoA into LPE and LPC (46). By contrast, in this study, it was found that human LPLAT10 transfers C26:0-CoA into LPC but not LPE. To address this discrepancy, we performed docking simulations of PC with C26:0 moiety and a 3D structural model of LPLAT10 obtained from AlphaFold2 (Supplemental Fig. S4A, B). The carbonyl carbon of the C26:0 moiety was located close (5.5 Å) to a histidine residue (H129) at the active site, and the C26:0-carbon chain was folded into a groove consisting of two helical structures (residues 134-138 and 158-165) (Supplemental Fig. S4C, D). By contrast, the trimethylamine moiety of the polar head was located between two tryptophan residues, 3.9 Å and 3.8 Å away from W242 and W244, respectively (Fig. 5A). To examine the roles of tryptophan residues in the substrate recognition of LPLAT10, we prepared membrane fractions expressing LPLAT10 with a mutation at the tryptophan residue (W244A) (Fig. 5B). We found that the activity of transferring C26:0-CoA to 2-acyl-LPC was almost completely lost in the membrane fractions expressing the W244A mutant (Fig. 5C). The activity of transferring C16:0-*d*_4_-CoA into 2-acyl-LPC in the mutant membrane fraction was almost comparable to that in the control membrane fraction (mock). The activity of transferring C16:0-*d*_4_-CoA into 1-acyl-LPC was almost equivalent to that of the control (Fig. 5C). Taken together, these results indicate that cation-π interactions between the trimethylamine moiety and tryptophan residues can be involved in the substrate selectivity of the enzyme; LPLAT10 prefers LPC as an acceptor substrate when it transfers C26:0-CoA. The W-X-W motif identified in this study is conserved in LPLAT8/LPCAT1, LPLAT9/LPCAT2, and LPLAT10/LPCAT4/LPEAT2/AGPAT7, belonging to the AYTL subfamily and could be important in the recognition of LPC as an acceptor substrate (Fig. 5A).

### Chemical synthesis of tetradeuterated C26:0 FFA (C26:0 FFA-*d*_4_)

**Fig. 6:**
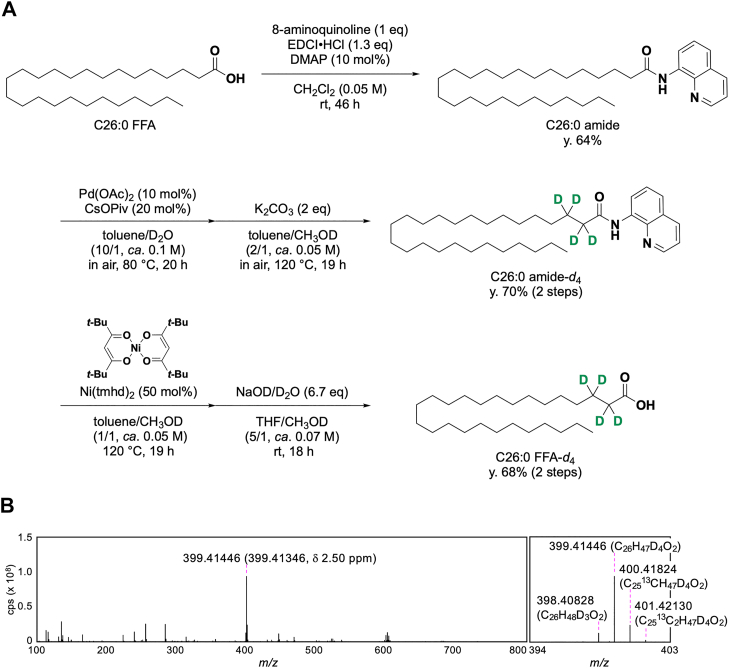
Chemical synthesis of C26:0 FFA-*d*_4_. A: C26:0 FFA was converted to the corresponding 8-aminoquinoline amide. Deuterium incorporation was first achieved selectively at the β-position under palladium catalysis, followed by the introduction of two deuterium atoms at the α-position using potassium carbonate. Finally, the resulting tetradeuterated amide was converted in two steps to furnish C26:0 FFA-*d*_4_. B: The ion spectra of the C26:0 FFA-*d*_4_ obtained by a high-resolution mass spectrometer. *ca*., circa; eq, equivalent; y., yield.

In X-ALD patients, VLCFA-CoAs that have not been degraded by β-oxidation in peroxisomes are incorporated into phospholipids, sphingolipids, and neutral lipids, such as TGs and CEs (48). To obtain a tracer molecule for analyzing the metabolic pathway of VLCFA in cells lacking both ABCD1 and LPLAT10, we chemically synthesized C26:0 FFA with four deuterium atoms introduced at the α- and β-positions, in accordance with a modified version of a procedure that we previously developed for straight-chain FA (Fig. 6A) (30). Notably, owing to the poor solubility of VLCFA, the solvent systems in each step of the tetradeuteration process were adapted to enhance the reaction efficacy. Initially, the C26:0 FFA was converted to the corresponding C26:0 amide with 8-aminoquinoline. This amide moiety was used for the following selective deuteration reactions. The Pd-catalyzed deuteration selectively afforded the β-deuterated C26:0 amide-*d*_2_, followed by the K_2_CO_3_-mediated reaction leading to selective α-deuteration. The latter step in CH_3_OD did not proceed well under our previously established reaction conditions, but it was found that toluene was effective as a cosolvent to improve its solubility. Thus, high deuterium content was achieved in the formation of the α,β-deuterated C26:0 amide-*d*_4_ (99% at the α-position and 95% at the β-position). The obtained deuterated amide was then converted to the corresponding C26:0 FFA-*d*_4_ in the following two steps. The CH_3_OD-toluene solvent system was also effective for the Ni-catalyzed transformation of C26:0 amide-*d*_4_ to C26:0 methyl ester-*d*_4_. The saponification of the methyl ester into the desired C26:0 FFA-*d*_4_ was accomplished in NaOD-D_2_O solution. The difference between the measured exact mass of the obtained C26:0 FFA-*d*_4_ and the mass calculated theoretically is 2.50 ppm, which confirms that the C26:0 FFA was properly tetradeuterated. Via this procedure, C26:0 FFA-*d*_4_ was efficiently produced, which was used for the following experiments.

### C26:0 is mainly metabolized into sphingolipids in HeLa cells lacking ABCD1 and LPLAT10

**Fig. 7:**
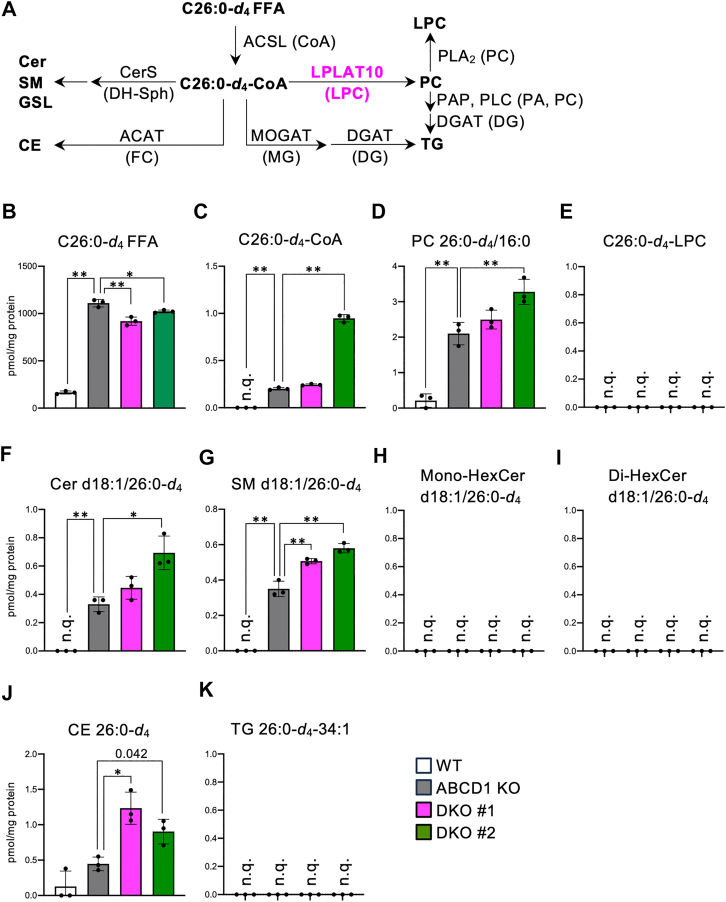
Metabolic analysis of C26:0 FFA-*d*_4_ in ABCD1 and LPLAT10-DKO HeLa cells. A: Schematic illustration of the possible metabolic pathway of exogenously administered C26:0 FFA-*d*_4_. Note that the C26:0-*d*_4_ moiety is subjected to hydrolysis by lipases, resulting in C26:0 FFA-*d*_4_. B–K: The amounts of C26:0 FFA-*d*_4_ (B) and lipid species with C26:0-*d*_4_ moiety (C–K) present within HeLa cells. Data represent the mean ± SD; statistical analysis was performed using one-way ANOVA, followed by the Dunnett’s test (vs. ABCD1 KO). *∗P* < 0.01, *∗∗P* < 0.001. ACAT, acyl-CoA:cholesterol acyltransferase; CerS, ceramide synthase; DG, diacylglycerol; DGAT, diacylglycerol O-acyltransferase; DH-Sph, dihydrosphingosine; FC, free cholesterol; GSL, glycosphingolipid; MG, monoacylglycerol; MOGAT, monoacylglycerol O-acyltransferase; PAP, phosphatidic acid phosphatase; PLA_2_, phospholipase A_2_; PLC, phospholipase C.

We treated ABCD1 and LPLAT10-DKO HeLa cells with the C26:0 FFA-*d*_4_ for 24 h and analyzed the amounts of FFA, acyl-CoA, TG, CE, phospholipids, and sphingolipids, such as Cer synthase, hexosylceramide (HexCer), and SM containing C26:0-*d*_4_ (Fig. 7A). The amounts of C26:0 FFA-*d*_4_ and lipid species with a C26:0-*d*_4_ acyl moiety were significantly increased in ABCD1-KO cells compared with those in wild-type cells, confirming that C26:0 FFA-*d*_4_ taken up into cells is not degraded properly because of the ABCD1 deficiency (Fig. 7B–D, F, G, J). The amount of C26:0 FFA-*d*_4_ was significantly reduced in ABCD1 and LPLAT10-DKO cells (Fig. 7B), whereas the amounts of Cer and SM, as well as CE with C26:0-*d*_4_ moiety (Cer d18:1/26:0-*d*_4_ and SM d18:1/26:0-*d*_4,_ CE 26:0-*d*_4_), were significantly increased in these cells (Fig. 7F, G, and J). These results indicate that exogenously administered C26:0 FFA-*d*_4_ is converted into C26:0-*d*_4_-CoA, after which it is preferably transferred into dihydrosphingosine and converted to Cer and SM or free cholesterol in ABCD1 and LPLAT10-DKO cells. Mono-HexCer and di-HexCer, as other sphingolipid species, as well as TG and LPC, were also analyzed in this study, but no lipid species containing C26:0-*d*_4_ moiety were found (Fig. 7E, H, I, and K). Glycolipid molecules, including gangliosides, are present within cells in smaller quantities than SM and Cer are. Since the amounts of glycosphingolipids and neutral lipids accumulated in each cell vary greatly, it is necessary to analyze them in other cells and tissues using a more sensitive measurement system. Unexpectedly, the amount of PC with C26:0-*d*_4_ moiety (PC 26:0-*d*_4_/16:0) did not decrease but instead increased in a line of ABCD1- and LPLAT10-DKO cells compared with that in ABCD1-KO cells (Fig. 7D). One possible cause of this is that LPLAT8-transferred C26:0-*d*_4_-CoA significantly accumulated in DKO #2 cells into 2–16:0-LPC (Fig. 7C). Previously, it has been shown that C26:0 FFA added exogenously are converted into C26:0-CoA much less efficiently than in the case of C18:1 FFA into C18:1-CoA (20). Thus, the synthetic pathways of C26:0-*d*_4_-PC that do not involve LPLAT10 may be highly dependent on the concentration of C26:0-*d*_4_-CoA.

## Discussion

In this study, we demonstrated that LPLAT10 transfers VLCFA-CoA, including C26:0-CoA, into 2-acyl-LPC to synthesize PC with C26:0 moiety and thereby contributes to the production of C26:0-LPC in fibroblasts from X-ALD patients and ABCD1-KO HeLa cells. LPLAT10 is characterized by its broad substrate selectivity for acyl-CoA species as donor molecules. Therefore, under pathological conditions in which VLCFA-CoA accumulates because of ABCD1 dysfunction, LPLAT10 mainly contributes to the synthesis of PC with VLCFA moiety.

LPLAT10 has been shown to catalyze the incorporation of long-chain fatty acyl groups into the *sn*-2 position of 1-acyl LPC. In the present study, however, higher levels of C26:0-LPC accumulated in ABCD1 KO cells than in ABCD1/LPLAT10 DKO cells (Fig. 3A). One possible explanation for this observation is that LPLAT10 may have a limited ability to recognize *sn*-1 C26:0 LPC as an acceptor molecule. Further characterization of substrate specificity with respect to the fatty acyl moieties of acceptor molecules, including carbon chain length and degree of unsaturation, is expected to clarify these issues in future studies.

The molecular mechanism responsible for generating C26:0-LPC from C26:0-PC is still unclear. Recently, it has been suggested that cPLA2α-PLA2G4A contributes to the production of LPC with a VLCFA moiety in the HDL fraction from *Abcd1*-deficient astrocytes in mice (49). However, it is still unclear whether C26:0-LPC in plasma from X-ALD patients is derived from that produced intracellularly or extracellularly. Detailed analysis of various molecules with PLA_2_ activity present inside and outside cells should reveal the entire mechanism by which C26:0-LPC is produced in X-ALD patients.

LPLAT8, which has high homology with LPLAT10, was shown to transfer C16:0-CoA to 2-acyl-type LPC, but its activity against C26:0-CoA was significantly lower than that of LPLAT10 (Fig. 4) (40). LPLAT9, like LPLAT8, is a molecule highly homologous to LPLAT10, but no activity against C26:0-CoA was detected at all (Fig. 4A). LPLAT9 has been reported to introduce acetyl groups into lysoPAF (1-*O*-alkyl-glycerophosphocholine) or LPC, suggesting that LPLAT9 may strictly recognize fatty acid chain length (43). In contrast, LPLAT10 transfers saturated fatty acyl-CoA with various chain lengths (C_16_–C_30_) as well as 20:4-CoA and a hydroxyoctadecadienoic acid as a PUFA and an oxylipin, respectively (Fig. 4) (30, 46). Intriguingly, Kawana *et al.* demonstrated that LPLAT10 transfers various PUFA-CoA species, including C22:6-CoA, to 2-acyl-LPC both in vitro and in vivo, indicating that LPLAT10 is characterized by its ability to utilize acyl-CoA molecules with diverse chain lengths and degrees of unsaturation as donor substrates. Previous studies have demonstrated that human LPLAT10 and mouse LPLAT10 exhibit enzymatic activity to incorporate long-chain fatty acyl-CoA, such as C18:1-CoA and C20:4-CoA, into LPC and LPE as acceptor molecules (46, 47). Intriguingly, LPLAT10 transfers C26:0-CoA into LPC but not into LPE (Fig. 4E). The 3D structural model of LPLAT10 and MD simulation with PC 26:0/16:0, as well as biochemical analysis using a mutant (W244A), suggests that the W-X-W motif in LPLAT10 recognizes trimethylamine groups of PC via cation-π bonds (Fig. 5). Given that previous reports described interactions between molecules containing trimethylamine groups and tryptophan residues via cation-π bonds in nicotinic acetylcholine receptors, the W-X-W motif may have important roles in strengthening the interaction between LPC and LPLAT10 and evenly LPLAT8 and LPLAT9 belonging to the AYTL subfamily (50).

In this study, we attempted to clarify the LPLAT10-dependent or -independent metabolic pathways of VLCFAs using C26:0 FFA-*d*_4_. Unexpectedly, the results indicate that the metabolic behavior of exogenously administered C26:0 FFA-*d*_4_ may differ from that of the VLCFA already present in cells (Fig. 7). Within cells, VLCFA-CoA is produced directly from long-chain fatty acyl-CoA such as C16:0-CoA, through fatty acid elongation reactions in X-ALD patients (3, 51), whereas exogenous very long-chain FFA need to be converted to VLCFA-CoA by long-chain acyl-CoA synthetases (ACSLs) or very long-chain acyl-CoA synthetases (ACSVLs) (52). Accumulating evidence from previous studies indicates that ACSL1 and ACSL5 primarily utilize FFAs with carbon chain lengths of C_16_–C_18_, whereas ACSL3, ACSL4, and ACSL6 are capable of utilizing PUFAs as substrates (53). In contrast, FATP1, FATP3, FATP5, and FATP6 primarily utilize FFAs with carbon chain lengths of C_16_–C_18_, whereas FATP2 and FATP4 (also known as ACSVL1 and ACSVL4, respectively) are classified as very long-chain acyl-CoA synthetases and preferentially activate fatty acids with chain lengths ≥C_22_ (52). Elucidating the substrate specificity of these fatty acid-activating enzymes toward C_26_ in detail may provide insights into the metabolic fate of exogenously added C26:0 FFA and its contribution to cellular toxicity.

Newborn screening for X-ALD using VLCFA-LPC as a clinical marker makes a major contribution to presymptomatic diagnosis and early medical intervention. However, psychological stress is caused by false-positive results as well as by variants of uncertain significance, and there are also problems associated with other diseases besides X-ALD being detected in such screening (15). Moreover, the direct causal relationship between X-ALD pathology and VLCFA-LPC itself remains unclear, although PC with VLCFA moiety has been suggested to be toxic to neurons in the brain (54). It is hoped that this study will lead to elucidation of the mechanism by which VLCFA-LPC is produced in patients and will contribute to the accurate diagnosis of X-ALD and clarification of the pathological role of VLCFA-LPC.

## Data Availability

The data that support the findings of this study are available from the corresponding authors upon reasonable request.

## Supplemental Data

This article contains supplemental data.

## Conflict of Interest

The authors declare that they have no conflicts of interest with the contents of this article.

## Supplemental Data

Paper_Sup_Final
