# Supplementary material for: Phosphatidylcholine with C26:0 moiety, a precursor of a diagnostic marker for X-ALD, is synthesized by LPLAT10/LPEAT2
Source: J Lipid Res. 2025 Dec 30;67(2):100973. doi: 10.1016/j.jlr.2025.100973 (PMC12859765; doi:10.1016/j.jlr.2025.100973)
Supplement: Paper_Sup_Final [file mmc1.docx]

**Synthesis of C26:0 FFA-*d*_4_**

Step 1. Condensation of the C26:0 FFA and 8-aminoquinoline

C26:0 FFA (595.1 mg, 1.5 mmol), 8-aminoquinoline (216.3 mg, 1.5 mmol, 1.0 eq), 1-ethyl-3-(3-dimethylaminopropyl)carbodiimide monohydrochloride (EDCI‧HCl, 372.7 mg, 1.95 mmol, 1.3 eq), DMAP (18.3 mg, 0.15 mmol, 10 mol%), and CH_2_Cl_2_ (30 mL, 0.05 M) were added to a three-neck flask under an argon atmosphere and stirred at room temperature for 46 h. The reaction progress was monitored by TLC analysis. The reaction was quenched with saturated aqueous NH_4_Cl solution and washed with brine. The aqueous layer was extracted with CH_2_Cl_2_ several times, and the combined organic layers were dried over Na_2_SO_4_, filtered, and evaporated under reduced pressure. The obtained material was purified by silica gel flash column chromatography (eluent: CH_2_Cl_2_/*n*-hexane = 50/50) to afford the C26:0 amide (486.1 mg, 64% yield) as a white powder.

Step 2. β-Selective deuteration of the C26:0 amide

C26:0 amide (39.9 mg, 0.08 mmol), palladium acetate (1.7 mg, 8 µmol, 10 mol%), and cesium pivalate (3.5 mg, 0.015 mmol, 20 mol%) were added to a 50 mL screw-capped tube under an air atmosphere. To the reaction mixture was added toluene (0.8 mL, 0.1 M), followed by sonication to dissolve the C26:0 amide. Subsequently, D_2_O (0.08 mL, 1 M) was added to the mixture. The resultant mixture was stirred at 80 °C for 20 h and then cooled to room temperature, diluted with AcOEt, and washed with brine. The organic layer was dried over Na_2_SO_4_, filtered, and evaporated under reduced pressure. The resultant residue was filtered through a pad of silica gel (eluent: CH_2_Cl_2_) to afford the crude C26:0 amide-*d*_2_ (46.5 mg) as a white powder. This material was used in the next step without further purification.

Step 3. α-Selective deuteration of the C26:0 amide-*d*_2_

The obtained C26:0 amide-*d*_2_ (46.5 mg), potassium carbonate (24.5 mg, 0.17 mmol, 2.0 eq), toluene (1.77 mL, 0.05 M), and methanol-*d*_1_ (CH_3_OD, 5.9 mL, 0.1 M) were added to a 50 mL screw-capped tube under an air atmosphere. The reaction mixture was stirred at 120 °C for 19 h. Then, the reaction mixture was cooled to room temperature, diluted with AcOEt, and washed with brine. The organic layer was dried over Na_2_SO_4_, filtered, and evaporated under reduced pressure to afford the C26:0 amide-*d*_4_ (28.1 mg, 70% yield in 2 steps). The deuteration rates were determined by ^1^H NMR analysis as 99% for the α-position and 95% for the β-position.

Step 4. Transformation of the C26:0 amide-*d*_4_ to the C26:0 methyl ester-*d*_4_

C26:0 amide-*d*_4_ (97% deuteration at the α-position and 95% deuteration at the β-position, 100.0 mg, 0.19 mmol), nickel bis(2,2,6,6-tetramethyl-3,5-heptanedionate) (40.3 mg, 0.095 mmol, 50 mol%), toluene (1.9 mL, 0.1 M), and methanol-*d*_1_ (CH_3_OD, 1.9 mL, 0.1 M) were added to a 50 mL screw-capped tube. The reaction mixture was stirred at 120 °C for 19 h. Then, it was cooled to room temperature, diluted with AcOEt, and washed with brine. The organic layer was dried over Na_2_SO_4_, filtered, and evaporated under reduced pressure. The crude residue was roughly purified through a pad of silica gel (eluent: AcOEt) to afford the C26:0 methyl ester-*d*_4_, which was used for the next step without further purification.

Step 5. Saponification of C26:0 methyl ester-*d*_4_ to the C26:0 FFA-*d*_4_

C26:0 methyl ester-*d*_4_ obtained in Step 4, THF (2.15 mL, 0.09 M), methanol-*d*_1_ (CH_3_OD, 0.43 mL, 0.4 M), and NaOD, 40 w/w% solution in D_2_O (0.18 mL) were added to a 50 mL round-bottomed flask. After stirring the reaction mixture at room temperature for 18 h, the reaction was quenched with 1 M HCl and the mixture was extracted with AcOEt. Then, the organic layer was dried over Na_2_SO_4_, filtered, and evaporated under reduced pressure. The crude residue was filtered through a pad of silica gel (eluent: CH_2_Cl_2_/*n*-hexane = 50/50) to afford the C26:0 FFA-*d*_4_ (51.5 mg, 68% yield in 2 steps).

Supplemental Table S1 The sequence of siRNA used in the screening

| Gene | Sequence (5’ to 3’) | ID # |
| --- | --- | --- |
| LPLAT1/AGPAT1 | CGUCUAAUGCUGCUCCACATT | s7 |
| LPLAT2/AGPAT2 | GUUCUUCACUUCAGGAACATT | s223130 |
| LPLAT3/AGPAT3 | GACUGAUAGGAGUAACUGATT | s32330 |
| LPLAT4/AGPAT4 | GUAUAUGACUGUACACUCATT | s32333 |
| LPLAT5/AGPAT5 | CAAAGAUAAGAUGCUUAUATT | s30737 |
| LPLAT6/LCLAT1/AGPAT8 | CAGCAAGUCUCGAAGUAAUTT | s48421 |
| LPLAT7/LPGAT1 | CAACGAUAGCUUAUCCCAATT | s19258 |
| LPLAT8/LPCAT1 | GGACCUGCCUAAUUACCUUTT | s36575 |
| LPLAT9/LPCAT2 | GCCAGUAAUAAAGUCAGCCCUGAAA | Custom |
| LPLAT10/LPCAT4/LPEAT2/AGPAT7 | GCAGCAUUGUGGAUGUGGATT | s48553 |
| LPLAT11/MBOAT7 | GCGCAGUAUAUCUACAAGATT | s35614 |
| LPLAT12/MBOAT5/LPCAT3 | CAAGUUCCUUGGAAAUAAATT | s19801 |
| LPLAT13/MBOAT2 | GUAUUUGAGUUACAACUGUTT | s43426 |
| LPLAT14/MBOAT1 | GAGGUGAACUGGAAGCGAATT | s45849 |
| GPAT1/GPAM | GUAGGAAAGUUUAUCCAGUTT | s224484 |
| GPAT2 | CCUCCUCUCUACUCACAAATT | s45488 |
| GPAT3 | GAAGGAACUUGCAUCAACAAUACUU | Custom |
| GPAT4/AGPAT6 | CCCUGUUGCUAUCAAGUAUTT | s44067 |

Supplemental Table S2 The sequence of oligonucleotide pairs for each guide RNA

| Gene | Sequence (5’ to 3’) |
| --- | --- |
| LPLAT10/LPCAT4/LPEAT2/AGPAT7 | CACCGGCCACGAACGCGAATCCGG |
|  | AAACCCGGATTCGCGTTCGTGGCC |
| LPLAT6/LCLAT1/AGPAT8 | CACCTTGTGGTGCTTCTGAACCCA |
|  | AAACTGGGTTCAGAAGCACCACAA |
| GPAT2 | CACCCCAGGTCATAGAGCAGCTGC |
|  | AAACGCAGCTGCTCTATGACCTGG |
| GPAT3 | CACCTAGGATCTTCATGTAGATCT |
|  | AAACAGATCTACATGAAGATCCTA |

Supplemental Table S3 The sequence of oligonucleotide pairs and restriction enzymes (RE) for PCR-RE-based screening

| Gene | For 1st PCR (5’ to 3’) | For 2nd PCR (5’ to 3’) | RE |
| --- | --- | --- | --- |
| LPLAT10 | GACTGTGTGCCACAACGG | AACGGGGTGCTAGGCCTG | *AccIII* |
|  | CAATGACAGGAACGGAAAGG | TTCTCAGCTCGGGACACAAC |  |
| LPLAT6 | TACTCTCTTCTGGGAAGCTG | GCAACAAATGGATGATGTG | *NcoI* |
|  | ACCCAAAGCTTTTAGAGTCC | CCTGCTTTATGAAGTACGTGC |  |
| GPAT2 | CTTTTCCTGCCCCCAGAG | CTCTCCCTGGACAGCTCTGA | *PvuII* |
|  | GACTGCCTGCACCACAAAC | TCCTCCAGGAAGATGAGCAG |  |
| GPAT3 | ATGGAGGGCGCAGAGCTG | GGAAGATCCTTTCCACCTG | *BglII* |
|  | GCATCCCTCCCGGCACTC | CACCTCTAAAGTTTTCACTAGG |  |

Supplemental Table S4 The composition and the gradient conditions of mobile phases used in LC-MS/MS analyses

| Lipid class | Composition  (A: hydrophilic, B: hydrophobic) | Gradient (min, % of B line) | Flow  (µL/  min) |
| --- | --- | --- | --- |
| PC, PE, PS, PI, PG, SM, TG, CE | A: ACN/MeOH/H_2_O = 2/2/1*^a,b^*  B: Isopropanol(v/v/v) *^b^* | 0-5, 0%; 5-9, 0 to 20%; 9-59, 20 to 65%; 59-60, 65 to 75%; 60-70, 75%; 70-74, 75 to 0% | 280 |
| Cer, HexCer, SM | A: ACN/MeOH/H_2_O = 19/19/1*^b^*  B: Isopropanol(v/v/v) *^b^* | 0-3, 3%; 3-24, 3 to 40%; 24-25, 40 to 70%; 25-32, 70%; 32 -33, 70 to 3%; 33-40, 3% | 200 |
| FFA | A: 5mM NH_4_HCO_2_*^c^*  B: 5mM NH_4_HCO_2_ in isopropanol/H_2_O = 19/1*^c^* | 0-1, 30%; 1-11, 30 to 95%; 11-15, 95%; 15-16, 95 to 30%; 16-18.5, 30% | 200 |
| Acyl-CoA | A: 5mM NH_4_HCO_2_*^c^*  B: 5mM NH_4_HCO_2_ in isopropanol/H_2_O = 19/1*^c^* | 0-2, 40%; 2-15, 40 to 100%; 15-17, 100%; 17-18, 100 to 40%; 18-20, 40% | 200 |
| LPC | A: 5mM NH_4_HCO_2_ in ACN/H_2_O = 4/6*^d^*  B: 5mM NH_4_HCO_2_ in isopropanol/H_2_O = 19/1*^d^* | 0-4, 20%; 4-20, 20 to 100%; 20-25, 100%; 25-26, 100 to 20%; 26-30, 20% | 200 |

*^a^* ACN, acetonitrile; MeOH, methanol

*^b^* Supplemented with 0.1% NH_4_HCO_2_ & 0.028% NH_3_

*^c^* Adjusted to pH 9.0 with ammonium hydroxide

*^d^* Adjusted to pH 4.0 with formic acid

Supplemental Table S5 The conditions of ion source used for quantitative analysis

| Lipid class | CUR*^a^* | CAD*^a^* | ISV*^a^* | Temp*^a^* | IG1*^a^* | IG2*^a^* |
| --- | --- | --- | --- | --- | --- | --- |
| PC, PE, PS, PI, PG, SM, TG, CE | 10 | 9 | 5500 | 200 | 40 | 80 |
| Cer, Mono-HexCer, Di-HexCer, SM | 40 | 10 | 5000 | 300 | 40 | 80 |
| Acyl-CoA | 40 | 9 | 5500 | 300 | 40 | 80 |
| FFA | 40 | 10 | −4500 | 300 | 40 | 80 |
| LPC | 40 | 9 | −4500 | 500 | 40 | 80 |
| PC, PE, SM (MS^3^ for structural analysis) | 40 | High | −4500 | 200 | 40 | 80 |
| TG (EPI for structural analysis) | 10 | High | 5500 | 200 | 40 | 80 |

*^a^* CUR, Curtain gas (L/min); CAD, Collision Gas; , ISV, Ion spray voltage (V); Temp, Temperature (°C); IG1, Ion source gas 1 (psi); IG2, Ion source gas 2 (psi)

Supplemental Table S6 The parameters of selected reaction monitoring (SRM) mode for each lipid class

| Lipid class | Q1*^a^* | Q3*^b^* | DP*^c^* (V) | EP*^c^* (V) | CE*^c^* (V) | CXP*^c^*  (V) |
| --- | --- | --- | --- | --- | --- | --- |
| PC | [M+H]^+^ | 184 | 26 | 10 | 77 | 12 |
| PE | [M+H]^+^ | M−140 | 101 | 10 | 33 | 14 |
| PS | [M+H]^+^ | M−184 | 121 | 10 | 31 | 16 |
| PI | [M+NH_4_]^+^ | M−259 | 81 | 10 | 37 | 16 |
| PG | [M+NH_4_]^+^ | M−171 | 41 | 10 | 35 | 16 |
| SM | [M+H]^+^ | 184 | 1 | 10 | 35 | 12 |
| Cer | [M+H]^+^ | 264 | 60 | 10 | 40 | 13 |
| Mono-HexCer | [M+H]^+^ | 264 | 60 | 10 | 40 | 13 |
| Di-HexCer | [M+H]^+^ | 264 | 60 | 10 | 60 | 13 |
| SM | [M+H]^+^ | 184 | 60 | 10 | 60 | 13 |
| TG | [M+NH_4_]^+^ | M−255 (C16:0-moiety) | 26 | 10 | 22 | 12 |
|  | [M+NH_4_]^+^ | M−367 (C24:0-moiety) |  |  |  |  |
|  | [M+NH_4_]^+^ | M−395 (C26:0-moiety) |  |  |  |  |
| CE | [M+NH_4_]^+^ | 369 | 26 | 10 | 22 | 12 |
| LPC | [M+HCOO]^−^ | M−222 | −26 | −10 | −40 | −15 |
| FFA | [M−H]^−^ | M−1 | −26 | −10 | −5 | −15 |
| Acyl-CoA | [M+H]^+^ | M−488 | 1 | 10 | 52.5 | 12 |

*^a^* Precursor ions selected in quadrupole 1 (Q1)

*^b^* The value of *m/z* selected as product ions

*^c^* DP, declustering potential; EP, entrance potential; CE, collision energy; CXP, collision cell exit potential; ET, excitation time

Supplemental Table S7 The parameters of enhanced product ion scanning (EPI) and MS/MS/MS (MS^3^) mode for each lipid class

| Lipid class | mode | Q1 | Q3/EPI | DP (V) | EP (V) | CE (V) | CES*^a^* | AF2*^a^* | Q3 Entry  Barrier  (V) | Fill Time  (ms) | ET*^a^* (ms) |
| --- | --- | --- | --- | --- | --- | --- | --- | --- | --- | --- | --- |
| PC | MS^3^ | [M+HCOO]^−^ | M−15 | −26 | −10 | −40 | 0 | 0.2 | 8 | D*^a^* | 25 |
| PE | MS^3^ | [M−H]^−^ | M−1 | −26 | −10 | −10 | 0 | 0.2 | 8 | D*^a^* | 25 |
| SM | MS^3^ | [M+HCOO]^−^ | M−15 | −26 | −10 | −40 | 0 | 0.2 | 8 | D*^a^* | 25 |
| TG | EPI | [M+NH_4_]^+^ | - | 26 | 10 | 22 | 0 | - | 8 | D*^a^* | - |

*^a^* CES, Collision Energy Spread; AF2, Excitation energy; ET, Excitation time; D, Dynamic

Supplemental Table S8 The internal standards used for quantitative analysis

| Lipid class | Internal standard | Q1 (*m/z*) | Q3 (*m/z*) |
| --- | --- | --- | --- |
| PC | PC 15:0/18:1-*d*_7_ | 753 | 184 |
| PE | PE 15:0/18:1-*d*_7_ | 711 | 570 |
| PS | PS 15:0/18:1-*d*_7_ | 755 | 570 |
| PI | PI 15:0/18:1-*d*_7_ | 847 | 570 |
| PG | PG 15:0/18:1-*d*_7_ | 759 | 570 |
| SM | SM d18:1/18:1-*d*_9_ | 738 | 184 |
| Cer | Cer d18:1/16:0-*d*_31_ | 569 | 264 |
| Mono-HexCer | Glucosyl-Ceramide d18:1/16:0-*d*_3_ | 703 | 264 |
| Di-HexCer | Lactosyl-Ceramide d18:1/17:0 | 876 | 264 |
| SM | SM d18:1/16:0-*d*_31_ | 734 | 184 |
| TG | TG 17:0-17:0-17:1*-d*_5_ | 869 | 582 |
| CE | CE 18:1-*d*_7_ | 675 | 376 |
| LPC | C17:0-LPC | 554 | 269 |
| FFA | C16:0-*d*_4_-FFA | 259 | 259 |
| Acyl-CoA | C16:0-*d*_31_-CoA | 1037 | 530 |

Supplemental Table S9 The quantity of each PL species in the X-ALD fibroblasts*^a^*

| Cell | Control | | X-ALD #1 | | X-ALD #2 | | X-ALD #3 | |
| --- | --- | --- | --- | --- | --- | --- | --- | --- |
| *guide RNA* | *control* | *LPLAT10* | *control* | *LPLAT10* | *control* | *LPLAT10* | *control* | *LPLAT10* |
| PC 32:0 | 219,429.5 | 215,713.2 | 171,861.0 | 181,541.5 | 179,412.1 | 203,969.5 | 276,444.3 | 248,583.9 |
| PC 32:1 | 251,379.9 | 289,476.1 | 213,784.2 | 235,183.1 | 218,237.4 | 259,302.0 | 278,548.4 | 276,963.0 |
| PC 32:2 | 35,196.4 | 39,846.8 | 25,835.7 | 27,790.2 | 31,383.1 | 37,301.5^**^ | 33,277.4 | 32,604.0 |
| PC 32:3 | 2,898.8 | 3,745.3^*^ | 2,855.7 | 3,056.2 | 4,434.3 | 4,995.3 | 4,721.9 | 5,136.5 |
| PC 34:0 | 91,580.2 | 102,167.5 | 61,223.2 | 60,386.6 | 71,872.1 | 75,519.9 | 123,226.8 | 95,586.0 |
| PC 34:1 | 425,296.8 | 478,706.5 | 468,320.0 | 509,925.4 | 404,844.0 | 486,046.1 | 556,993.8 | 541,827.9 |
| PC 34:2 | 237,266.2 | 266,732.9 | 199,590.6 | 200,762.7 | 212,116.2 | 244,314.6 | 246,941.7 | 219,527.2 |
| PC 34:3 | 28,716.8 | 33,770.1^*^ | 23,134.8 | 22,048.3 | 27,034.7 | 28,201.1 | 30,006.6 | 27,245.4 |
| PC 34:4 | 7,145.4 | 9,296.1^**^ | 7,974.9 | 9,562.2 | 13,173.4 | 13,709.6 | 8,944.9 | 8,818.8 |
| PC 34:5 | 781.7 | 816.1 | 778.4 | 834.9 | 1,075.2 | 1,131.1 | 636.0 | 677.0 |
| PC 34:6 | 189.0 | 68.0 | 118.4 | 94.8 | 0.0 | 0.0 | 0.0 | 0.0 |
| PC 36:0 | 3,857.1 | 3,795.0 | 2,777.8 | 2,752.7 | 4,884.4 | 5,034.3 | 7,057.6 | 5,658.2^*^ |
| PC 36:1 | 209,569.9 | 263,753.0^*^ | 232,212.7 | 265,503.6 | 230,392.8 | 281,471.9^*^ | 346,211.0 | 339,027.2 |
| PC 36:2 | 295,400.0 | 360,567.6^*^ | 287,236.7 | 265,980.0 | 337,614.5 | 370,800.4 | 358,115.5 | 290,339.9 |
| PC 36:3 | 161,272.1 | 194,987.1^*^ | 139,948.1 | 136,047.9 | 128,404.5 | 126,586.5 | 155,418.5 | 127,334.6 |
| PC 36:4 | 137,963.2 | 184,727.3^**^ | 157,807.7 | 202,916.7 | 107,259.5 | 133,796.9^*^ | 160,967.9 | 171,889.3 |
| PC 36:5 | 48,116.3 | 50,283.1 | 43,346.8 | 52,298.2 | 29,235.5 | 34,735.1^*^ | 35,048.8 | 36,516.8 |
| PC 36:6 | 4,291.0 | 4,030.2 | 3,634.7 | 3,908.5 | 3,297.1 | 3,584.0 | 3,119.0 | 3,069.6 |
| PC 36:7 | 484.8 | 592.9 | 699.3 | 670.4 | 590.5 | 618.3 | 1,138.5 | 908.8 |
| PC 38:0 | 2,397.0 | 2,180.6 | 1,913.4 | 2,031.9 | 1,265.9 | 1,205.7 | 1,896.8 | 1,662.7 |
| PC 38:1 | 7,964.5 | 7,489.2 | 5,522.2 | 5,238.3 | 7,187.9 | 5,945.4^*^ | 9,548.8 | 7,249.4 |
| PC 38:2 | 12,616.4 | 11,046.8 | 12,323.5 | 8,086.7 | 21,578.9 | 13,698.7^**^ | 15,768.6 | 8,546.2^**^ |
| PC 38:3 | 32,270.2 | 41,288.7^*^ | 35,242.9 | 38,718.9 | 36,166.7 | 24,876.4 | 43,172.6 | 39,183.0 |
| PC 38:4 | 120,271.1 | 173,196.4^**^ | 151,912.1 | 207,036.2 | 107,310.4 | 143,478.9^**^ | 193,397.5 | 184,494.0 |
| PC 38:5 | 179,806.4 | 209,107.2^*^ | 179,662.6 | 189,764.5 | 136,961.4 | 146,404.1 | 182,184.4 | 165,143.8 |
| PC 38:6 | 60,301.6 | 64,163.6 | 48,229.8 | 48,714.1 | 45,870.0 | 47,298.5 | 54,659.6 | 47,968.6 |
| PC 38:7 | 7,434.5 | 6,981.3 | 5,298.6 | 4,853.8 | 5,749.0 | 5,712.6 | 6,753.7 | 5,433.6 |
| PC 38:8 | 306.9 | 348.2 | 325.5 | 277.9 | 351.7 | 358.9 | 436.6 | 418.8 |
| PC 40:0 | 516.0 | 518.4 | 655.0 | 655.7 | 627.3 | 484.1^*^ | 928.3 | 676.3^*^ |
| PC 40:1 | 1,020.6 | 997.3 | 1,059.9 | 932.6 | 1,617.2 | 1,286.2^**^ | 1,626.7 | 1,155.3^*^ |
| PC 40:2 | 1,181.6 | 1,025.5 | 1,200.1 | 747.0 | 2,422.4 | 1,413.1^**^ | 1,865.0 | 988.7^**^ |
| PC 40:3 | 1,131.3 | 1,615.9^**^ | 1,061.0 | 1,042.5 | 1,120.7 | 932.0^*^ | 2,190.6 | 1,926.5 |
| PC 40:4 | 3,100.7 | 3,905.5^*^ | 3,126.3 | 3,410.5 | 2,861.2 | 3,192.3^*^ | 4,947.3 | 4,777.6 |
| PC 40:5 | 39,820.2 | 52,897.1^**^ | 39,288.2 | 40,328.8 | 43,113.0 | 44,246.7 | 65,822.8 | 58,597.9 |
| PC 40:6 | 27,180.4 | 33,390.2^**^ | 21,751.9 | 18,895.9 | 33,080.6 | 28,277.7^**^ | 29,104.3 | 23,104.1 |
| PC 40:7 | 14,976.4 | 18,441.9^*^ | 14,549.6 | 11,838.4 | 24,484.5 | 22,827.1 | 18,880.7 | 15,789.3 |
| PC 40:8 | 3,775.8 | 4,846.0^*^ | 3,149.4 | 2,659.3 | 3,589.3 | 3,465.2 | 3,247.5 | 2,762.8 |
| PC 40:9 | 424.0 | 531.1^*^ | 411.5 | 409.4 | 495.8 | 543.7 | 358.3 | 391.3 |
| PC 40:10 | 77.3 | 76.4 | 59.3 | 23.9 | 89.6 | 95.9 | 37.1 | 36.6 |
| PC 42:0 | 258.5 | 248.5 | 1,021.7 | 845.6 | 1,181.9 | 782.3^**^ | 1,691.2 | 1,082.0^**^ |
| PC 42:1 | 789.2 | 896.8^*^ | 1,356.6 | 1,171.4 | 2,264.1 | 1,681.7^**^ | 2,139.3 | 1,532.5 |
| PC 42:2 | 720.2 | 727.7 | 968.4 | 659.5^*^ | 1,700.8 | 1,074.5^**^ | 1,299.7 | 814.4^*^ |
| PC 42:3 | 499.1 | 607.9 | 458.1 | 337.7 | 1,323.0 | 854.7^**^ | 787.3 | 542.8 |
| PC 42:4 | 1,369.0 | 1,825.0^*^ | 780.9 | 643.0 | 2,200.8 | 1,240.3^**^ | 1,850.3 | 1,323.7 |
| PC 42:5 | 3,531.4 | 4,121.2^*^ | 2,375.2 | 1,745.7 | 4,939.3 | 2,998.7^**^ | 3,752.4 | 2,735.4 |
| PC 42:6 | 3,922.7 | 4,430.8^*^ | 2,462.5 | 1,811.1 | 5,372.7 | 3,544.6^**^ | 3,768.9 | 2,365.5^*^ |
| PC 42:7 | 1,995.6 | 1,855.3 | 1,738.0 | 1,111.7 | 3,571.7 | 1,992.3^*^ | 1,860.3 | 1,289.1 |
| PC 42:8 | 1,876.8 | 2,301.1^*^ | 1,978.8 | 1,481.7 | 3,444.3 | 2,420.0^**^ | 2,706.8 | 1,939.6 |
| PC 42:9 | 3,409.9 | 4,512.2 | 3,604.2 | 3,169.8 | 4,800.8 | 4,483.2 | 4,299.4 | 3,708.7 |
| PC 42:10 | 2,328.8 | 2,652.2^*^ | 2,038.4 | 1,725.7 | 2,311.5 | 1,890.2^*^ | 2,211.7 | 1,770.8 |
| PC 42:11 | 250.2 | 243.1^*^ | 173.7 | 130.0 | 208.3 | 156.9 | 150.6 | 126.9 |
| PC 44:0 | 22.8 | 40.8 | 337.0 | 259.0 | 469.6 | 283.5^**^ | 606.9 | 377.1^*^ |
| PC 44:1 | 236.6 | 191.2 | 1,283.0 | 795.8 | 2,524.1 | 1,174.9^**^ | 2,085.8 | 1,076.6^**^ |
| PC 44:2 | 274.2 | 252.7^**^ | 793.7 | 494.2 | 2,096.3 | 1,075.2^**^ | 1,345.4 | 733.4^**^ |
| PC 44:3 | 197.9 | 211.8 | 355.9 | 169.3^**^ | 953.9 | 530.1^**^ | 572.8 | 380.8^*^ |
| PC 44:4 | 156.1 | 131.8 | 276.8 | 220.1 | 262.6 | 127.9^**^ | 258.1 | 147.2^**^ |
| PC 44:5 | 292.6 | 259.7 | 505.1 | 289.1 | 871.9 | 390.2^**^ | 689.7 | 304.2^**^ |
| PC 44:6 | 544.5 | 552.8 | 236.8 | 86.4^*^ | 1,257.3 | 545.3^**^ | 296.9 | 287.4 |
| PC 44:7 | 406.9 | 454.3 | 288.2 | 147.6^*^ | 1,030.3 | 386.3^**^ | 435.1 | 148.3^**^ |
| PC 44:8 | 625.4 | 722.4 | 544.2 | 304.8^*^ | 1,173.4 | 558.5^**^ | 852.3 | 325.6^**^ |
| PC 44:9 | 1,182.6 | 1,381.1^*^ | 936.5 | 655.6 | 1,762.7 | 1,079.6^**^ | 1,376.7 | 894.2^*^ |
| PC 44:10 | 943.7 | 1,172.2^*^ | 642.9 | 637.2 | 1,161.9 | 967.7^*^ | 1,086.5 | 1,076.4 |
| PC 44:11 | 779.8 | 1,003.7^*^ | 631.0 | 522.5 | 1,297.1 | 1,194.5 | 963.7 | 839.8 |
| PC 44:12 | 182.6 | 204.0 | 168.0 | 133.6 | 261.5 | 159.6^**^ | 206.1 | 150.2 |
| PC 46:0 | 0.0 | 0.0 | 70.5 | 58.5 | 125.7 | 83.4^**^ | 128.6 | 81.6^*^ |
| Cell | Control | | X-ALD #1 | | X-ALD #2 | | X-ALD #3 | |
| *guide RNA* | *control* | *LPLAT10* | *control* | *LPLAT10* | *control* | *LPLAT10* | *control* | *LPLAT10* |
| PC 46:1 | 0.0 | 0.0 | 283.0 | 163.9 | 686.2 | 294.7^**^ | 445.5 | 205.7^**^ |
| PC 46:2 | 0.0 | 0.0 | 201.5 | 124.6 | 744.5 | 338.8^**^ | 371.1 | 201.6^**^ |
| PC 46:3 | 0.0 | 0.0 | 139.4 | 92.4 | 414.1 | 192.6^**^ | 247.3 | 89.3^**^ |
| PC 46:4 | 90.1 | 61.1^*^ | 668.1 | 305.5^**^ | 1,067.3 | 243.1^**^ | 950.7 | 251.2^**^ |
| PC 46:5 | 148.6 | 136.7 | 512.1 | 313.0 | 1,082.2 | 334.5^**^ | 766.2 | 309.5^**^ |
| PC 46:6 | 109.3 | 99.9 | 161.4 | 87.5^*^ | 467.3 | 229.7^*^ | 236.3 | 126.7^**^ |
| PC 46:7 | 0.0 | 0.0 | 18.0 | 0.0 | 59.6 | 11.6^*^ | 32.8 | 30.2 |
| PC 46:8 | 0.0 | 26.5 | 0.0 | 0.0 | 10.5 | 18.4 | 25.9 | 0.0 |
| PC 46:9 | 230.0 | 233.8 | 136.8 | 86.2 | 540.6 | 191.1^**^ | 265.4 | 116.0^**^ |
| PC 46:10 | 196.3 | 233.7 | 108.7 | 42.1 | 436.7 | 157.3^**^ | 131.2 | 89.2 |
| PC 46:11 | 89.5 | 115.9 | 38.3 | 36.8 | 210.8 | 102.9^**^ | 103.6 | 46.3 |
| PE 32:0 | 179.6 | 157.9^**^ | 98.3 | 101.1 | 159.2 | 143.3 | 225.3 | 158.0 |
| PE 32:1 | 324.7 | 306.4 | 219.9 | 227.6 | 279.2 | 298.0 | 323.1 | 267.6 |
| PE 32:2 | 24.7 | 10.4 | 0.0 | 0.0 | 0.0 | 0.0 | 0.0 | 0.0 |
| PE 34:0 | 218.3 | 237.5^**^ | 132.0 | 126.5 | 242.1 | 212.6 | 291.9 | 226.1 |
| PE 34:1 | 3,144.3 | 3,340.0 | 2,445.6 | 2,610.3 | 3,524.6 | 3,752.1 | 3,770.1 | 3,058.8 |
| PE 34:2 | 880.4 | 831.0 | 660.4 | 612.5 | 721.1 | 702.0 | 846.0 | 732.7 |
| PE 34:3 | 58.4 | 55.0 | 31.2 | 20.0 | 42.9 | 52.3 | 37.5 | 23.9 |
| PE 36:1 | 3,985.9 | 4,838.0^**^ | 3,633.2 | 3,802.4 | 5,024.9 | 5,568.7 | 5,884.1 | 5,271.4 |
| PE 36:2 | 3,768.5 | 4,213.3^*^ | 3,098.7 | 2,945.3 | 3,966.4 | 4,132.1 | 3,605.2 | 3,192.7 |
| PE 36:3 | 939.6 | 1,040.4 | 720.3 | 719.8 | 767.3 | 778.8 | 822.1 | 754.5 |
| PE 36:4 | 745.8 | 779.9 | 636.3 | 681.1 | 476.5 | 540.9 | 706.6 | 599.2 |
| PE 36:5 | 173.0 | 137.0^**^ | 131.3 | 137.8 | 98.5 | 103.0 | 114.0 | 104.7 |
| PE 38:1 | 132.1 | 146.1 | 110.6 | 116.3 | 165.9 | 160.9 | 171.1 | 165.4 |
| PE 38:2 | 142.5 | 169.0^*^ | 131.5 | 122.4 | 188.4 | 192.4 | 160.7 | 152.0 |
| PE 38:3 | 1,548.4 | 1,562.3 | 1,560.3 | 1,772.7 | 1,582.4 | 1,500.4 | 1,993.6 | 1,960.7 |
| PE 38:4 | 6,108.9 | 8,691.3^**^ | 5,279.7 | 5,892.4 | 4,014.4 | 4,606.2 | 7,504.4 | 6,638.3 |
| PE 38:5 | 3,692.0 | 3,512.1 | 2,872.5 | 2,994.8 | 2,458.4 | 2,583.3 | 3,220.2 | 2,770.8 |
| PE 38:6 | 1,653.7 | 1,440.7^*^ | 1,120.5 | 1,249.8 | 866.8 | 892.2 | 1,544.0 | 1,350.5 |
| PE 38:7 | 181.2 | 156.3 | 120.9 | 126.0 | 161.0 | 160.5 | 177.3 | 144.7 |
| PE 40:1 | 72.8 | 90.8^**^ | 51.7 | 38.7 | 99.5 | 109.1 | 71.1 | 61.9 |
| PE 40:2 | 63.4 | 83.4 | 40.4 | 36.8 | 116.8 | 121.3 | 75.3 | 78.3 |
| PE 40:3 | 70.1 | 90.3^**^ | 60.6 | 67.2 | 86.3 | 91.2 | 123.3 | 116.6 |
| PE 40:4 | 981.9 | 1,420.4^**^ | 785.9 | 893.1 | 1,188.8 | 1,337.7 | 2,566.6 | 2,435.7 |
| PE 40:5 | 1,070.3 | 1,401.6^**^ | 846.5 | 889.7 | 1,783.8 | 2,028.5 | 2,525.5 | 2,342.6 |
| PE 40:6 | 1,415.7 | 1,692.4^**^ | 1,091.0 | 1,275.5 | 2,332.0 | 2,572.4 | 3,167.4 | 2,811.9 |
| PE 40:7 | 945.9 | 1,084.4^*^ | 774.4 | 892.7 | 1,167.1 | 1,275.8 | 1,184.3 | 1,165.1 |
| PE 40:8 | 29.1 | 56.9 | 14.7 | 30.0 | 42.7 | 46.6 | 50.0 | 23.8 |
| PE 42:1 | 132.1 | 145.0 | 121.7 | 130.7 | 193.1 | 180.1 | 161.3 | 145.2 |
| PE 42:2 | 33.7 | 54.6 | 17.4 | 35.9 | 73.6 | 81.3 | 58.8 | 22.0 |
| PE 42:3 | 0.0 | 0.0 | 0.0 | 0.0 | 35.2 | 0.0^**^ | 0.0 | 0.0 |
| PE 42:4 | 0.0 | 101.9^**^ | 0.0 | 17.2 | 75.9 | 99.4 | 94.2 | 86.6 |
| PE 42:5 | 95.6 | 129.8^**^ | 64.2 | 48.0 | 120.3 | 122.1 | 129.0 | 118.2 |
| PE 42:6 | 90.2 | 112.5^**^ | 54.1 | 40.1 | 141.1 | 141.7 | 108.0 | 107.1 |
| PE 42:7 | 37.0 | 69.5 | 17.6 | 33.6 | 98.5 | 107.7 | 70.6 | 73.5 |
| PE 42:8 | 22.0 | 46.3 | 0.0 | 0.0 | 44.8 | 51.7 | 15.8 | 18.0 |
| PE 44:1 | 10.6 | 10.7 | 46.9 | 47.7 | 73.2 | 65.8 | 66.0 | 59.4 |
| PE 44:2 | 0.0 | 0.0 | 0.0 | 0.0 | 53.4 | 54.8 | 0.0 | 0.0 |
| PE 44:5 | 0.0 | 0.0 | 0.0 | 0.0 | 47.7 | 42.8 | 0.0 | 0.0 |
| PG 34:1 | 403.0 | 421.2 | 262.7 | 317.9 | 320.8 | 346.4 | 359.1 | 334.1 |
| PG 36:1 | 200.8 | 228.7 | 148.4 | 171.3 | 447.1 | 474.2 | 296.5 | 253.7 |
| PG 36:2 | 151.8 | 81.0^*^ | 103.4 | 111.0 | 71.7 | 82.7^*^ | 239.9 | 201.2 |
| PI 32:0 | 123.4 | 69.1^**^ | 75.3 | 66.8 | 65.7 | 53.1 | 188.3 | 128.3^*^ |
| PI 32:1 | 165.3 | 127.5^*^ | 119.6 | 98.8 | 138.6 | 112.8^*^ | 197.7 | 140.2 |
| PI 34:0 | 268.1 | 190.1^**^ | 279.8 | 185.3 | 310.2 | 295.8 | 590.3 | 429.8^*^ |
| PI 34:1 | 1,856.8 | 1,578.2 | 1,395.8 | 1,123.0 | 1,714.6 | 1,513.7 | 2,614.3 | 1,992.8 |
| PI 34:2 | 631.8 | 589.5 | 494.7 | 411.3 | 431.5 | 410.4 | 778.4 | 668.5 |
| PI 34:3 | 60.9 | 47.3 | 48.0 | 0.0^**^ | 57.3 | 47.0 | 76.3 | 73.0 |
| PI 36:1 | 2,124.8 | 2,347.1 | 1,705.1 | 1,410.8 | 2,711.7 | 2,811.8 | 2,443.5 | 1,999.5 |
| PI 36:3 | 969.3 | 1,036.9 | 757.5 | 749.8 | 678.9 | 660.5 | 1,035.0 | 953.6 |
| PI 36:4 | 1,436.6 | 1,335.9 | 1,057.5 | 1,106.8 | 967.2 | 935.8 | 1,579.5 | 1,343.6 |
| PI 36:5 | 104.0 | 72.4^*^ | 66.6 | 66.5 | 47.4 | 38.9 | 90.5 | 81.2 |
| PI 38:2 | 71.6 | 58.9^*^ | 43.6 | 33.7 | 95.2 | 88.7 | 70.7 | 64.8 |
| PI 38:3 | 2,318.4 | 3,695.6 | 2,154.0 | 2,717.3 | 3,267.1 | 3,480.4 | 1,908.9 | 1,603.3 |
| PI 38:4 | 35,519.5 | 39,306.5 | 27,760.9 | 30,379.6 | 29,641.6 | 28,738.6 | 37,381.6 | 33,748.6 |
| PI 38:5 | 6,176.8 | 6,353.5 | 4,308.9 | 4,615.1 | 3,521.8 | 3,440.5 | 6,204.0 | 5,776.6 |
| PI 38:6 | 651.3 | 530.1^*^ | 394.6 | 392.3 | 383.7 | 338.4 | 499.9 | 417.2 |

| Cell | Control | | X-ALD #1 | | X-ALD #2 | | X-ALD #3 | |
| --- | --- | --- | --- | --- | --- | --- | --- | --- |
| *guide RNA* | *control* | *LPLAT10* | *control* | *LPLAT10* | *control* | *LPLAT10* | *control* | *LPLAT10* |
| PI 40:3 | 76.2 | 80.7 | 45.3 | 43.2 | 139.3 | 117.5^**^ | 85.0 | 85.4 |
| PI 40:4 | 1,075.9 | 1,598.0^**^ | 805.6 | 822.8 | 1,954.2 | 1,982.9 | 1,515.7 | 1,336.5 |
| PI 40:5 | 3,166.7 | 3,477.8 | 2,148.3 | 2,263.1 | 5,084.9 | 4,897.5 | 3,425.7 | 3,254.0 |
| PI 40:6 | 792.4 | 790.5 | 482.4 | 502.1 | 1,122.1 | 1,115.1 | 593.9 | 496.2 |
| PI 40:7 | 358.0 | 375.3 | 256.0 | 240.3 | 220.5 | 222.9 | 323.8 | 322.0 |
| PI 42:5 | 24.0 | 39.6 | 0.0 | 0.0 | 46.1 | 49.3 | 31.5 | 0.0 |
| PI 42:6 | 0.0 | 0.0 | 0.0 | 0.0 | 9.4 | 0.0 | 0.0 | 0.0 |
| PI 42:8 | 31.4 | 59.7 | 0.0 | 0.0 | 0.0 | 0.0 | 0.0 | 0.0 |
| PI 42:9 | 0.0 | 10.1 | 0.0 | 0.0 | 0.0 | 0.0 | 0.0 | 0.0 |
| PS 32:0 | 100.3 | 119.2 | 67.2 | 38.2 | 142.4 | 116.9 | 92.8 | 81.4 |
| PS 32:1 | 109.9 | 93.3 | 61.1 | 67.6 | 84.1 | 79.4^*^ | 53.7 | 42.6 |
| PS 34:0 | 226.3 | 386.3^*^ | 192.1 | 130.7 | 251.1 | 170.7 | 275.4 | 186.6 |
| PS 34:1 | 2,754.9 | 2,927.7 | 2,046.8 | 2,165.1 | 1,926.1 | 1,789.4 | 2,090.5 | 1,808.4 |
| PS 34:2 | 201.6 | 191.7 | 131.6 | 114.4 | 99.5 | 88.5 | 107.3 | 85.8 |
| PS 36:1 | 13,778.7 | 17,647.1 | 14,908.6 | 16,173.7 | 7,006.2 | 6,743.7 | 14,737.6 | 13,271.4 |
| PS 36:2 | 3,099.4 | 3,643.4 | 2,734.6 | 2,627.0 | 1,427.6 | 1,312.8 | 2,175.7 | 1,952.5 |
| PS 36:3 | 233.0 | 266.0 | 204.3 | 204.7 | 119.0 | 119.4 | 173.3 | 142.4 |
| PS 36:4 | 62.9 | 62.4 | 57.3 | 50.5 | 37.0 | 42.0 | 15.1 | 15.5 |
| PS 38:1 | 427.5 | 462.0 | 363.7 | 362.3 | 169.5 | 154.8 | 318.7 | 284.1 |
| PS 38:2 | 548.8 | 566.5 | 541.5 | 556.4 | 256.1 | 203.7 | 475.8 | 478.6 |
| PS 38:3 | 2,306.7 | 3,337.1^*^ | 2,447.3 | 2,613.0 | 1,260.1 | 1,214.5 | 2,925.1 | 2,391.4 |
| PS 38:4 | 2,637.5 | 3,499.4 | 2,388.2 | 2,960.8 | 1,314.7 | 1,276.5 | 2,168.9 | 1,998.6 |
| PS 38:5 | 401.8 | 399.0 | 317.2 | 359.8 | 208.3 | 208.2 | 231.0 | 208.3 |
| PS 38:6 | 219.9 | 144.8 | 148.8 | 173.1 | 96.7 | 93.6 | 180.6 | 145.5 |
| PS 40:1 | 713.5 | 716.0 | 628.4 | 660.1 | 278.0 | 255.0 | 546.3 | 493.9 |
| PS 40:2 | 304.7 | 337.6 | 293.3 | 334.0 | 150.5 | 127.0 | 279.1 | 246.0 |
| PS 40:3 | 75.4 | 55.6 | 85.3 | 83.0 | 50.5 | 16.6 | 31.9 | 38.9 |
| PS 40:4 | 1,079.4 | 1,418.1 | 975.7 | 1,015.2 | 678.5 | 637.2 | 2,792.8 | 2,383.0 |
| PS 40:5 | 1,916.9 | 2,129.3 | 1,867.4 | 1,921.0 | 2,497.8 | 2,271.2 | 6,675.8 | 5,690.6 |
| PS 40:6 | 2,054.3 | 1,954.7 | 1,845.4 | 2,094.5 | 2,333.5 | 1,983.4 | 6,095.5 | 5,157.3 |
| PS 40:7 | 88.6 | 87.4 | 77.3 | 79.6 | 83.5 | 65.4 | 155.8 | 143.8 |
| PS 42:1 | 394.6 | 363.3 | 497.8 | 532.7 | 174.1 | 160.4 | 395.1 | 384.3 |
| PS 42:2 | 284.6 | 257.7 | 328.1 | 346.5 | 149.8 | 140.9 | 256.0 | 281.5 |
| PS 42:3 | 91.2 | 77.5 | 93.1 | 97.0 | 44.2 | 38.2 | 86.7 | 82.3 |
| PS 42:4 | 122.6 | 142.3 | 91.8 | 81.7 | 78.3 | 64.2 | 151.6 | 133.3 |
| PS 42:5 | 143.6 | 156.8 | 96.5 | 101.6 | 101.7 | 78.5 | 190.8 | 172.0 |
| PS 42:6 | 130.4 | 128.6 | 63.9 | 71.3 | 91.4 | 72.6 | 144.4 | 116.0 |
| PS 42:7 | 10.4 | 0.0 | 0.0 | 0.0 | 9.1 | 0.0 | 0.0 | 15.5 |
| PS 44:1 | 0.0 | 0.0 | 61.3 | 41.4 | 0.0 | 0.0 | 40.0 | 40.8 |
| PS 44:2 | 0.0 | 0.0 | 16.7 | 15.3 | 10.2 | 0.0 | 15.3 | 16.2 |
| PS 44:3 | 0.0 | 10.9 | 18.7 | 13.3 | 11.7 | 0.0 | 30.9 | 18.7 |
| PS 44:4 | 0.0 | 0.0 | 0.0 | 0.0 | 0.0 | 0.0 | 18.1 | 36.9 |
| PS 44:5 | 0.0 | 0.0 | 0.0 | 0.0 | 28.4 | 12.8 | 68.9 | 46.3 |
| SM 32:0 | 473.1 | 396.2 | 450.9 | 407.6 | 281.8 | 280.1 | 544.1 | 436.8 |
| SM 32:1 | 3,125.4 | 3,353.1 | 2,623.9 | 2,610.2 | 2,150.5 | 2,151.2 | 2,389.0 | 2,277.9 |
| SM 32:2 | 59.6 | 66.4^*^ | 53.6 | 60.1 | 49.2 | 46.9 | 52.4 | 36.7 |
| SM 34:0 | 7,067.9 | 5,439.7^*^ | 7,919.3 | 6,959.2 | 2,866.2 | 2,703.5 | 9,866.2 | 7,862.1 |
| SM 34:1 | 40,145.1 | 38,587.5 | 40,913.7 | 44,907.0 | 23,787.0 | 27,206.1 | 41,433.0 | 41,662.2 |
| SM 34:2 | 5,102.6 | 5,621.4 | 4,053.0 | 4,226.5 | 3,003.4 | 3,101.9 | 4,085.2 | 3,772.8 |
| SM 34:3 | 21.5 | 31.2 | 0.0 | 0.0 | 30.0 | 40.2 | 0.0 | 0.0 |
| SM 36:1 | 4,886.5 | 4,862.0 | 4,439.2 | 5,061.5 | 2,623.2 | 3,222.7^*^ | 3,935.8 | 3,263.3 |
| SM 36:2 | 506.6 | 447.9 | 502.5 | 585.1 | 336.3 | 370.7 | 410.1 | 335.2 |
| SM 36:3 | 640.3 | 713.0 | 557.4 | 628.9 | 637.9 | 736.6 | 814.3 | 873.0 |
| SM 36:3 | 640.3 | 713.0 | 557.4 | 628.9 | 637.9 | 736.6 | 814.3 | 873.0 |
| SM 38:1 | 1,130.0 | 1,101.1 | 761.3 | 841.4 | 521.1 | 551.2 | 999.0 | 793.0 |
| SM 38:2 | 125.9 | 106.5 | 85.8 | 84.2 | 112.1 | 101.6 | 143.3 | 99.9^*^ |
| SM 38:3 | 174.1 | 152.9 | 183.5 | 208.9 | 117.8 | 134.2 | 172.5 | 197.7 |
| SM 40:0 | 434.2 | 278.6^*^ | 500.3 | 410.3 | 82.5 | 52.8 | 0.0 | 0.0 |
| SM 40:1 | 11,488.0 | 10,247.7 | 8,245.2 | 8,739.4 | 4,129.5 | 4,219.2 | 8,539.8 | 7,209.4 |
| SM 40:2 | 1,523.6 | 1,588.9 | 980.3 | 1,021.1 | 540.7 | 605.4^*^ | 964.7 | 970.3 |
| SM 42:0 | 449.2 | 288.6^*^ | 508.9 | 457.0 | 123.3 | 107.4 | 447.6 | 349.0 |
| SM 42:1 | 24,722.4 | 19,994.2 | 19,657.2 | 21,828.8 | 10,090.9 | 9,811.2 | 16,244.0 | 14,300.7 |
| SM 42:2 | 31,151.4 | 29,505.6 | 25,411.1 | 28,319.3 | 14,225.4 | 14,824.1 | 25,550.3 | 24,251.9 |
| SM 42:3 | 6,148.8 | 6,502.7 | 4,095.4 | 4,343.1 | 3,019.1 | 3,138.8 | 4,912.2 | 4,683.4 |
| SM 44:1 | 304.7 | 216.9 | 886.4 | 987.9 | 559.0 | 503.6^*^ | 873.6 | 771.0 |
| SM 44:2 | 0.0 | 0.0 | 1,279.8 | 1,401.3 | 1,008.4 | 969.4 | 1,335.9 | 1,257.1 |
| SM 44:3 | 401.8 | 377.9 | 459.2 | 471.1 | 487.1 | 466.6 | 547.7 | 527.8 |
| Cell | Control | | X-ALD #1 | | X-ALD #2 | | X-ALD #3 | |
| *guide RNA* | *control* | *LPLAT10* | *control* | *LPLAT10* | *control* | *LPLAT10* | *control* | *LPLAT10* |
| TG 16:0/32:0 | 249.0 | 275.2 | 276.4 | 281.9 | 207.1 | 215.0 | 332.2 | 286.1 |
| TG 16:0/34:1 | 93.0 | 101.8 | 54.6 | 56.4 | 87.7 | 81.5 | 105.0 | 91.3 |
| TG 16:0/36:2 | 32.3 | 43.8 | 21.0 | 21.9 | 50.0 | 47.9 | 40.3 | 37.8 |
| TG 16:0/40:0 | 6.5 | 6.7 | 7.7 | 9.6 | 6.2 | 7.8 | 8.3 | 8.5 |
| TG 16:0/42:0 | 3.0 | 3.1 | 3.4 | 3.8 | 3.5 | 3.9 | 4.7 | 5.2 |
| TG 16:0/42:1 | 0.6 | 1.3 | 0.3 | 0.0 | 1.4 | 1.7 | 1.3 | 0.8 |
| TG 16:0/44:1 | 0.0 | 0.0 | 0.3 | 0.0 | 1.8 | 1.8 | 1.4 | 0.9 |
| TG 16:0/44:2 | 1.2 | 0.4 | 1.1 | 3.6 | 0.0 | 0.0 | 0.0 | 0.0 |
| TG 16:0/46:2 | 0.0 | 0.0 | 0.0 | 0.0 | 1.0 | 1.1 | 0.0 | 0.0 |
| CE 14:0 | 1,339.7 | 190.5 | 1,037.2 | 1,080.8 | 0.0 | 0.0 | 0.0 | 0.0 |
| CE 16:0 | 173.5 | 188.0 | 243.0 | 284.5 | 205.6 | 235.3 | 264.6 | 231.3 |
| CE 16:1 | 234.0 | 226.3 | 354.9 | 389.7 | 266.9 | 297.8 | 297.6 | 271.4 |
| CE 18:0 | 65.8 | 70.0 | 95.9 | 121.0 | 96.5 | 110.1 | 74.6 | 67.8 |
| CE 18:1 | 1,754.7 | 2,002.6 | 2,675.4 | 3,292.7 | 2,871.7 | 3,305.3 | 2,315.5 | 2,313.1 |
| CE 18:2 | 1,631.7 | 1,570.3 | 2,237.1 | 2,717.9 | 1,696.1 | 2,080.6 | 1,642.3 | 1,506.9 |
| CE 18:3 | 426.7 | 392.7 | 622.0 | 718.9 | 533.7 | 578.7 | 403.6 | 398.8 |
| CE 20:1 | 94.4 | 110.5 | 157.0 | 191.3 | 194.2 | 284.8^*^ | 83.0 | 90.0 |
| CE 20:2 | 285.8 | 409.3^*^ | 540.8 | 555.5 | 931.9 | 1,206.7^*^ | 220.7 | 235.3 |
| CE 20:3 | 2,508.4 | 3,415.3 | 5,813.2 | 6,107.2 | 6,886.3 | 8,483.6^*^ | 2,016.1 | 2,164.2 |
| CE 20:4 | 4,484.3 | 5,288.4 | 8,531.3 | 9,361.4 | 9,385.7 | 11,623.7^**^ | 3,558.4 | 3,833.5 |
| CE 20:5 | 3,996.2 | 4,343.8 | 6,378.7 | 7,062.7 | 8,788.1 | 10,749.7^*^ | 2,388.7 | 2,641.7 |
| CE 22:1 | 51.9 | 69.0 | 93.5 | 114.8 | 132.5 | 175.9 | 14.7 | 17.9 |
| CE 22:2 | 73.0 | 110.0 | 142.0 | 141.3 | 289.5 | 391.2^*^ | 52.2 | 63.8 |
| CE 22:3 | 364.4 | 592.0 | 730.3 | 733.4 | 1,422.3 | 1,870.4^*^ | 229.9 | 288.7^*^ |
| CE 22:4 | 1,531.2 | 2,492.7^*^ | 3,175.6 | 3,461.3 | 6,382.7 | 7,901.2^**^ | 1,029.8 | 1,294.2^*^ |
| CE 22:5 | 5,700.6 | 7,948.6 | 10,149.3 | 10,973.7 | 16,179.6 | 19,146.3^*^ | 2,469.4 | 3,191.5^*^ |
| CE 22:6 | 7,976.8 | 10,042.9 | 10,095.1 | 10,909.7 | 17,419.8 | 19,465.5 | 4,098.7 | 4,650.8 |
| CE 24:0 | 62.8 | 61.3 | 109.5 | 139.1 | 84.8 | 101.5 | 77.8 | 74.3 |
| CE 24:1 | 141.7 | 180.3 | 339.1 | 404.5 | 400.6 | 588.3^*^ | 115.3 | 135.3 |
| CE 24:2 | 60.2 | 94.2 | 118.1 | 118.9 | 286.0 | 386.3 | 32.4 | 57.4 |
| CE 24:3 | 163.4 | 276.5 | 301.6 | 295.5 | 918.0 | 1,216.2^*^ | 100.7 | 123.4 |
| CE 24:4 | 474.9 | 796.2^*^ | 686.5 | 673.4 | 1,383.1 | 1,804.4^*^ | 213.8 | 277.6^*^ |
| CE 24:5 | 1,000.0 | 1,404.6 | 1,208.5 | 1,176.8 | 2,960.6 | 3,382.3 | 353.1 | 452.8^*^ |
| CE 24:6 | 470.0 | 669.0^*^ | 391.4 | 368.3 | 2,838.5 | 3,001.5 | 154.5 | 181.1 |
| CE 26:0 | 131.9 | 127.1 | 262.6 | 310.4 | 314.5 | 397.6 | 153.0 | 179.2 |
| CE 26:1 | 127.8 | 149.7 | 365.6 | 406.3 | 725.4 | 1,006.8^*^ | 146.6 | 185.3 |
| CE 26:2 | 62.9 | 96.8 | 147.8 | 144.5 | 546.1 | 736.2^*^ | 63.0 | 80.8 |
| CE 26:3 | 159.6 | 288.5^*^ | 228.0 | 209.9 | 1,149.8 | 1,433.0 | 119.6 | 138.5 |
| CE 26:4 | 333.7 | 592.1^*^ | 260.6 | 237.0 | 1,216.1 | 1,376.2 | 151.2 | 173.2 |
| CE 26:5 | 337.3 | 570.4^*^ | 219.9 | 201.4 | 2,036.5 | 2,111.6 | 123.6 | 132.4 |
| CE 26:6 | 319.9 | 451.6 | 142.1 | 120.3 | 1,932.1 | 1,861.8 | 69.9 | 82.5 |

*^a^*pmol/mg protein

^*^*p* < 0.05, ^**^*p* < 0.01 vs. *control guide RNA* (Student *t*-test)
